# Evaluation of the Anti-Inflammatory and Anti-Oxidative Effects of Therapeutic Human Lactoferrin Fragments

**DOI:** 10.3389/fbioe.2021.779018

**Published:** 2021-11-30

**Authors:** Yu Pan, Zhao Liu, Yijie Wang, Linshen Zhang, Niying Chua, Lei Dai, Jun Chen, Chun Loong Ho

**Affiliations:** ^1^ Department of Biomedical Engineering, Southern University of Science and Technology (SUSTech), Shenzhen, China; ^2^ School of Biological Sciences, Nanyang Technological University, Singapore, Singapore; ^3^ CAS Key Laboratory of Quantitative Engineering Biology, Chinese Academy of Sciences, Shenzhen Institute of Synthetic Biology, Shenzhen Institutes of Advanced Technology (SIAT), Shenzhen, China

**Keywords:** lactoferrin, anti-infammatory, protease-digested, non-malignant colonic fibroblasts cells, tNF-alpha

## Abstract

Chronic inflammation is considered a pressing health issue that needs resolving. Inflammatory disease such as inflammatory bowel disease requires a long-term medical regimen to prevent disease progression. Conventionally, lactoferrin is used to treat mild gastrointestinal tract and skin inflammation. Protease-digested lactoferrin fragments often exhibit improved therapeutic properties compared to full-length lactoferrin (flHLF). However, there are no studies on the use of protease-digested lactoferrin fragments to treat inflammation. Herein, we assess the anti-inflammatory properties of engineered recombinant lactoferrin fragments (rtHLF4, rteHLF1, and rpHLF2) on non-malignant colonic fibroblast cells and colorectal cancer cells. We found that rtHLF4 is 10 times more effective to prevent inflammation compared to flHLF. These results were investigated by looking into the reactive oxygen species (ROS) production, angiogenesis activity, and cellular proliferation of the treated cells. We have demonstrated in this study the anti-inflammatory properties of the flHLF and the various lactoferrin fragments. These results complement the anti-cancer properties of these proteins that were demonstrated in an earlier study.

## Introduction

Patients suffering from inflammatory bowel disease (IBD) are more at risk of developing colorectal cancer in the later stages of their lives (Barral et al., 2016). In the United States of America and Europe, more than 3 million patients are suffering from IBD, where the prevalence of IBD is estimated to exceed 0.3% in North American, Oceania, and European countries (Alatab et al., 2020). In the recent decade, the number of IBD patients in mainland China has shown a significant rise (Zhai et al., 2016). Depending on the severity, mild chronic IBD is usually treated using drugs such as aminosalicylates (Gisbert et al., 2007), corticosteroids (Waljee AK et al., 2016), immunomodulators (Biancone et al., 2014), and probiotic microbial regiment (Scaldaferri et al., 2013), whereas severe cases of IBD require open surgery to resect damaged tissues within the gastrointestinal tract (Maggiori and Panis, 2013). In line with this, there is an increasing need to develop less invasive treatments with higher efficacy to treat IBD.

Naturally occurring lactoferrin is an iron-binding glycoprotein released by inflamed tissues, functioning as the first line of defense in the mammalian host (Rosa et al., 2017). Lactoferrin is known to have anti-inflammatory (Conneely, 2001), antibacterial (Jahani et al., 2015), antioxidant (Ibuki et al., 2020), and anticarcinogenic (Rodrigues et al., 2009) properties. Certain protease-digested lactoferrin fragments were found to exhibit improved therapeutic activities (Gifford et al., 2005), where our recent research found that protease-digested recombinantly expressed full-length human lactoferrin (flHLF) showed improved anti-cancer properties (Pan et al., 2021). We identified three lactoferrin fragments (rtHLF4, rteHLF1, and rpHLF2) exhibiting improved anti-cancer activity. rtHLF4, in particular, showed up to 100-fold improved anti-cancer activity against various cancer cell lines compared to flHLF (Pan et al., 2021). In the study, rtHLF4 inhibits all the tested cancer cell lines, whereas rteHLF1 and rpHLF2 inhibit the growth of 75% of the tested cancer cell lines. In addition, only fragment rtHLF4 showed improved stability under human physiological temperature and pH (Pan et al., 2021). rtHLF4 exerts its anti-cancer properties by changing the expression levels of proteins involved in the death receptor pathway, mitochondrial outer membrane permeabilization, and P53-related pathway. We hypothesize that the anti-inflammatory activity of these lactoferrin fragments also contributes to the anti-cancer properties. This study follows up on our earlier investigation of the various lactoferrin fragments, studying the anti-inflammatory properties, in efforts to further develop these lactoferrin fragments as an IBD treatment and as a prevention of colorectal cancer progression.

## Materials and Methods

### Materials

All culturing media ingredients were purchased from Oxoid (Basingstoke, Hampshire, United Kingdom), Macklin (Shanghai, China), and Aladdin (Shanghai, China). Biochemical assay solutions were purchased from Aladdin, Dieckmann (Shenzhen, Guangdong, China), and Shanghai Lingfeng Chemical Reagent co., LTD. (Shanghai, China). I-TASSER server (https://zhanggroup.org//I-TASSER/) was used for the prediction of the 3D structure of the human lactoferrin. Other chemicals used are specified in the methods below.

### Gene Cloning

The rtHLF4, rteHLF1, and rpHLF2 genes were amplified by PCR from the full-length lactoferrin (Accession number: AAA59511) using primers in Supplementary Table S2. These fragment sequences were digested and ligated into *pET28b* expression vector. All these resulting constructs were confirmed by automated DNA sequencing using universal primers.

### Protein Purification

The plasmids (*pET28b-flHLF*, *pET28b-rtHLF4, pET28b-rteHLF1,* and *pET28b-rpHLF2*) were transformed into *E. coli* BL21 (DE3) cultured in lysogeny broth (LB) at 37°C, 220 rpm to OD_600_ = 0.6 followed by 0.5 mM isopropyl β-D-1-thiogalactopyranoside (IPTG) induction at 16°C, 160 rpm for 20 h. Cells were harvested, resuspended in lysis buffer (50 mM Na_2_HPO_4_, 300 mM NaCl, 10% glycerol, and 1 mM dithiothreitol, pH 7.5), and lysed using EmulsiFlex-C3 (Avestin). Expressed full-length lactoferrin and recombinant lactoferrin fragments (rtHLF4, rteHLF1, and rpHLF2) were purified by immobilized metal ion affinity chromatography nickel-nitrilotriacetic acid (IMAC Ni-NTA). The full-length lactoferrin and lactoferrin fragment rtHLF4 purification was followed by Hiload Superdex 200 size exclusion column chromatography (GE Healthcare). The lactoferrin fragment rteHLF1 and rpHLF2 purification followed by Hiload Superdex 75 size exclusion column chromatography (GE Healthcare). The purified protein was concentrated and stored at −80°C. We use a lipase assay kit (Acmec Biochemical, BC2340) to detect the percentage of LPS in full-length lactoferrin and various lactoferrin fragments. UTI89 was used as the positive control.

### Hemolytic Assay

Fresh blood was collected from healthy human volunteers (aged between 20 and 40 years old, devoid of any non-steroidal anti-inflammatory drugs for 2 weeks prior) and was mixed with an equal volume of sterilized Alsever solution (2% dextrose, 0.8% sodium citrate, 0.5% citric acid, and 0.42% sodium chloride). The blood was centrifuged for 5 min at 3,000 rpm. The concentrated cell stock was prepared by resuspending cell pellets in isosaline (0.9% w/v NaCl). Cell stock was diluted 10-fold in isosaline to achieve working concentrations. Various concentrations of the flHLF/rtHLF4/rteHLF1/rpHLF2 protein (100, 80, 50, 30, 10, 8, 5, 3, and 1 μM) were prepared in protein buffer, and to each concentration, 1 ml of phosphate buffer, 2 ml of hyposaline, and 0.5 ml of HRBC (human red blood cells) suspension were added. The cell suspensions were incubated at 37°C for 30 min and centrifuged at 3,000 rpm for 20 min. The hemoglobin content in the supernatant solution was estimated spectrophotometrically at 560 nm. Indomethacin (100 μg/ml) was used as the reference standard. The percentage of hemolysis was calculated by assuming the hemolysis produced by the control group as 100%. The HRBC membrane stabilization or protection percentage was calculated using the following formula: percent protection = 100 − [(OD of protein treated sample/OD of control) 100]. All experiments were conducted with the approval of the Institutional Review Board at the Southern University of Science and Technology, under the title of “Evaluation of the safety of therapeutic peptides by hemolysis” with approval number 2020SYG096.

### Cell Culture

Human non-cancer colon fibroblast CCD-841-CON and CCD-18co, HT29 human colorectal adenocarcinoma cells were obtained from the American Type Culture Collection (ATCC, Manassas, VA, USA). CCD-841-CON and CCD-18co were cultured in Eagle’s Minimum Essential Medium (WISENT, 320–006-CL), and HT29 cells were cultured in McCoy’s 5A medium (CELL COOK, CM 2002). All the cell culture media were supplemented with 1% antibiotics (penicillin/streptomycin) and 10% fetal bovine serum (v/v). All cell cultures were maintained at 37°C in a 5% CO_2_ atmosphere.

### Cell Viability Assay

CCD-841-CON and CCD-18co cell lines were cultured in EMEM medium supplemented with 1% antibiotics (penicillin/streptomycin) and 10% fetal bovine serum (v/v). HT29 cells were cultured in McCoy’s 5A medium supplemented with 1% antibiotics (penicillin/streptomycin) and 10% fetal bovine serum (v/v). These three cell lines were seeded in 96-well plates and incubated for 24 h to allow cell attachment. The cultures were incubated with full-length lactoferrin (flHLF), rtHLF4, rteHLF1, and rpHLF2 till 3-, 24-, and 72-h time points, and untreated wells were also included. The cell viability assay using CellTiter 96® Aqueous One Solution Kit (Promega, USA). Cell culture media was aspirated, and the cells were washed with phosphate-buffered saline (PBS) before incubating the cells with MTS for 1–4 h at 37°C in 5% CO_2_. Add 10% SDS to end the reaction. All wells were read at an absorbance wavelength of 490 nm. Relative cell viability was quantified with the fluorescence intensity in the control group considered as 100%. Three independent experiments were performed.

### Reactive Oxygen Species Generation Assay

CCD-841-CON and CCD-18co cell lines were cultured in normal EMEM medium with 10% fetal bovine serum (v/v). HT29 cells were cultured in McCoy’s 5A medium, which was supplemented with 10% fetal bovine serum (v/v). These three cell lines were seeded in 96-well plates and incubated for 24 h. Referring to the methods of Venencio’s article, the CCD-841-CON and CCD-18co cells were treated with TNF-α (10 ng/ml) for ROS induction, and all cultures were treated with flHLF, rtHLF4, rteHLF1, and rpHLF2, respectively for 24 h, and TNF-α (10 ng/ml) was used to induce ROS generation and inflammation in CCD-841-CON and CCD-18co (Venancio et al., 2017). The Reactive Oxygen Species Assay Kit (Beyotime, China) was used in ROS assay. Cells were washed twice with PBS before incubating with DCFH-DA for 20 min at 37°C, and the fluorescence intensity of each well was measured using a microplate reader at 488 nm excitation and 525 nm emission. Relative ROS generation was quantified with the fluorescence intensity in the control group considered as 100%. Three independent experiments were performed.

### Real-Time Reverse Transcription-Polymerase Chain Reaction

CCD-841-CON, CCD-18co, and HT29 cells were seeded in six-well plates and incubated for 24 h. Cells were treated with lactoferrin flHLF, rtHLF4, rteHLF1, and rpHLF2 for 24 h. In CCD-841-CON and CCD-18co cells, 10 ng/ml TNF-α was used to induce inflammation. Negative (cell only) and positive (TNF-α) controls were also performed. RNA in three wells was extracted using Eastep® Super Total RNA Extraction Kit (Promega, USA) as stated by the manufacturer. For the synthesis of cDNA, 1-μg RNAs of each sample was reversed transcribed to cDNA using the GoScriptTM Reverse Transcription System (Promega, USA), according to the manufacturer’s instructions. Subsequently, 20 ng of cDNAs was mixed with specific primers, and quantitative PCR (qPCR) was performed by using FasStart Universal SYBR Green Master (ROX) (Promega) using the ABI StepOne Plus Real-time PCR System (Applied Biosystems, Foster City, CA, USA). mRNA expression of nuclear factor kappa B (*NF-κB*), tumor necrosis factor-alpha (*TNF-α*), interleukin 1 beta (*IL-1β*), interleukin 6 (*IL-6*), interleukin 8 (*IL-8*), and cyclo-oxygenase-2 (*COX-2*) was analyzed using beta-actin (*ACTB*) as a reference gene. The primer sequence used in this study is provided in Supplementary Table S1. Data were calculated relative to the control group using the classic 2^−∆∆Ct^ method.

### Western Blot

Protein expression levels were analyzed by Western blot. Cells were seeded in six-well plates and incubated with lactoferrin flHLF, rtHLF4, rteHLF1, and rpHLF2 for 24 h. Cells were washed in PBS and lysed in RIPA buffer (0.6057 g Tris base, 0.877 g NaCl, 10 ml).

Nonident P-40, 5 ml of 10% Na-deoxycholate, 1 ml of 10% sodium dodecyl sulfate, pH set to 7.5, adjusted to 100 ml with H_2_O for 30 min before centrifugation and supernatant collection. Subsequently, 20 μl of whole-cell extracts were resolved by sodium dodecyl sulfate-polyacrylamide gel electrophoresis (SDS-PAGE) and transferred to polyvinylidene difluoride (PVDF) membranes. The membranes were blocked with 5% non-fat-milk and then incubated within primary antibodies: ACTB (Abcam, UK, ab213262), NF-ΚB (Abcam, UK, ab76302), TNF-α (Abcam, UK, ab183218), IL-1β (Abcam, UK, ab216995), IL-6 (Abcam, UK, ab233706), IL-8 (Abcam, UK, ab235584), and COX-2 (Abcam, UK, ab179800) at 4°C overnight. The membranes were then incubated with the secondary antibodies (Abcam, UK, ab6721) for 2 h. Blots were visualized by the enhanced chemiluminescence (ECL) method.

### Statistical Analysis

All data were presented from three independent experiments. Each experiment has three replicates. Statistical differences between groups were analyzed by Student’s *t*-test. Statistical significance thresholds are *p ≤ 0.05, **p ≤ 0.01, and ***p ≤ 0.001.

## Results and Discussion

### Expression and Characterization of Recombinant Engineered Lactoferrin Fragments

The recombinantly expressed lactoferrin fragments rtHLF4, rteHLF1, and rpHLF2 were based on our earlier published work (Pan et al., 2021). In that study, we observed that the rtHLF4 and flHLF protein could negate the indomethacin-induced hemolytic activity. This preliminary result suggests that on top of the anti-cancer properties, the lactoferrin fragments can further prevent inflammation, further reducing the chances of carcinogenesis of tumor cells.

**FIGURE 1:**
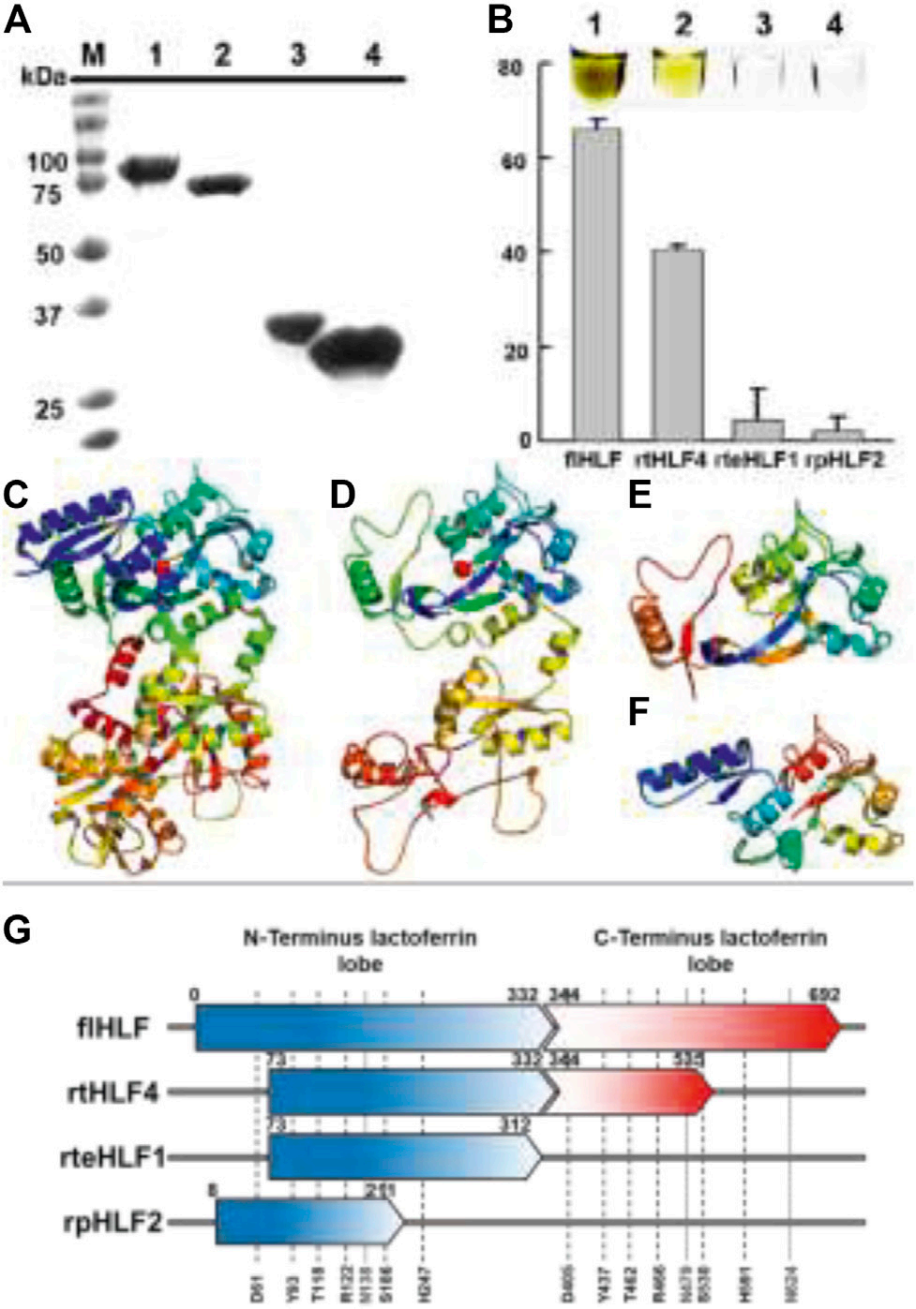
Expression of anti-inflammatory lactoferrin fragments. **(A)** SDS-PAGE of lactoferrin fragments (M, marker; 1, flHLF; 2, rtHLF4; 3, rteHLF1; 4, rpHLF2). **(B)** Percentage of ferric iron saturation of lactoferrin fragments (inset: 1, flHLF; 2, rtHLF4; 3, rteHLF1; 4, rpHLF2; samples prepared at 0.1 mM) (each group perform in triplicates). Model of lactoferrin fragments’ structure **(C)** flHLF; **(D)** rtHLF4; **(E)** rteHLF1; **(F)** rpHLF2. **(G)** The sequence length of lactoferrin-derived fragments compared to lactoferrin [iron-binding sites: 61, 122, 118, 186, 247, and 93 (indicated as black short dash); N-glycosylation sites: 138, 479, and 624 (indicated as gray dotted line)]. Anti-hemolytic assay of recombinant lactoferrin.

The soluble recombinantly expressed lactoferrin fragments were successfully purified (Figure 1A) and showed changes in the ferric binding ability supported by the changes in the color of the purified protein (Figure 1B). Based on the iron saturation assay, we found that rtHLF4, rteHLF1, and rpHLF2 showed lower levels of ferric occupancy than flHLF (Figure 1B). rtHLF4, showing a lighter brown hue than flHLF, showed approximately twofold lower in iron saturation, whereas the colorless purified rteHLF1 and rpHLF2 showed less than 5% in iron saturation (Figure 1B inset).

We hypothesize that the iron saturation is due to the truncation of the flHLF, perturbing the ferric binding domains within the lactoferrin structure. In order to understand the changes of rtHLF4, rteHLF1, and rpHLF2 iron saturation profile, we used the I-TASSER modeled structures of these three proteins that showed structural perturbation of the ferric ion-binding pocket resulting from the loss of critical residues within the N- and C-terminus regions of the proteins (Figures 1C–F). Closer investigation of the iron-binding conserved residues (comprising two tyrosines, a histidine and an aspartic acid, and two other carbonate-interacting residues that facilitate metal ion sequestration) showed that all the truncated sequences showed a loss of a single critical residue, resulting in the loss of the iron-binding ability (Lambert et al., 2005). Both rtHLF4 and rteHLF1 retain three conserved residues aside from D63, whereas rpHLF2 retains three conserved residues aside from H249 (Figure 1G, Supplementary Figure S1).

In order to ensure that the purified protein does not influence the inflammatory factors during testing, we found low levels of LPS contamination (<2.5%) from the purified proteins (Supplementary Figure S2).

We employed the indomethacin-hemolytic assay as a preliminary evaluation of the anti-inflammation properties of the four recombinant proteins. The indomethacin-hemolytic assay was previously used to evaluate plant extract’s anti-inflammatory and toxicity properties (Joseph et al., 2013), where indomethacin is used as a measure for membrane cytotoxicity mimicking the conditions of intracellular inflammation (Debnath et al., 2013).

**FIGURE 2:**
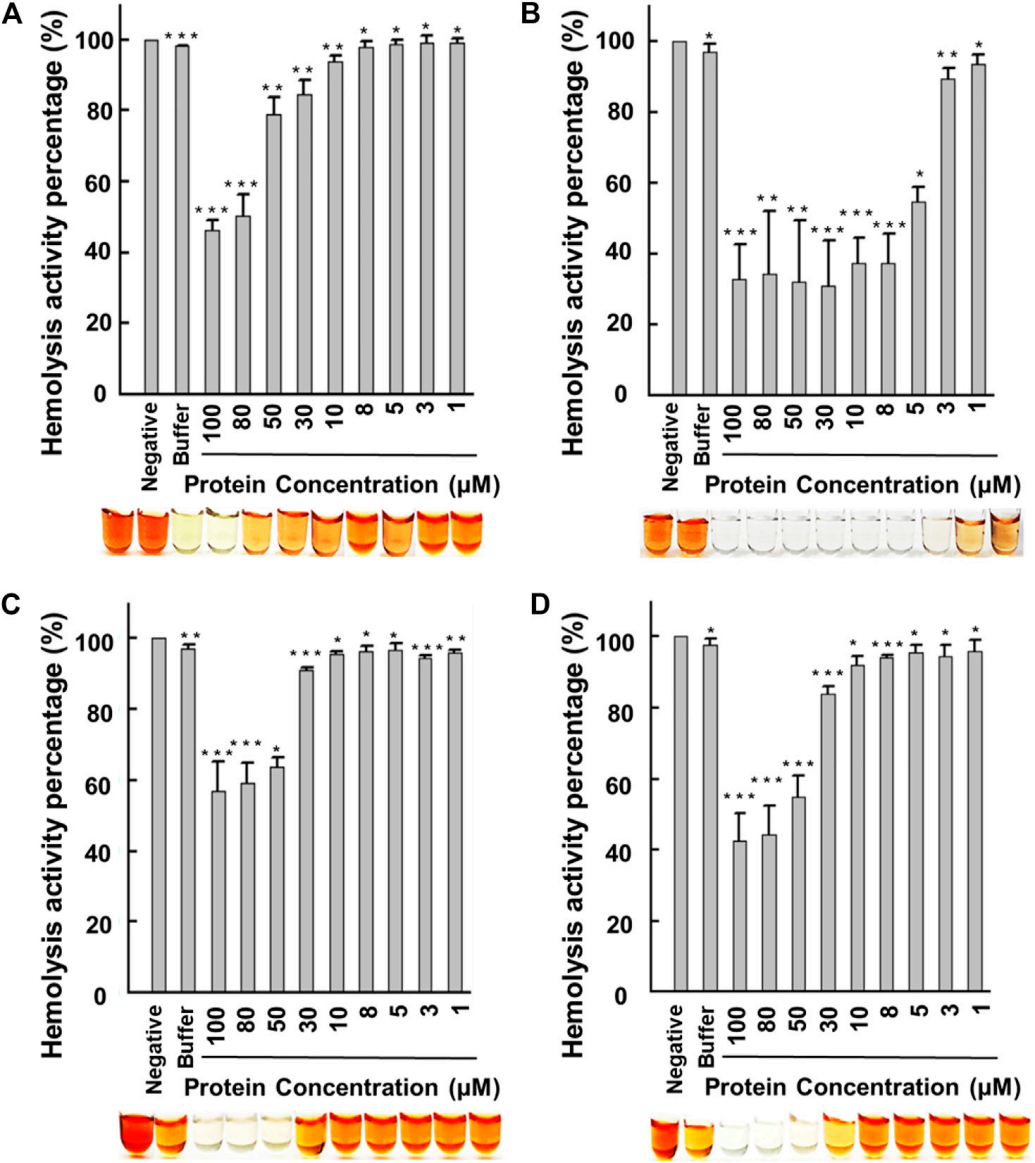
Human erythrocyte hemolytic assay using recombinant lactoferrin. Hemolysis activity of **(A)** flHLF; **(B)** rtHLF4; **(C)** rteHLF1; and **(D)** rpHLF2. (Inset: hemolytic results using the corresponding flHLF, rtHLF4, rteHLF1, and rpHLF2 concentration; *n* = 10, negative control, PBS) (*p ≤ 0.05, **p ≤ 0.01, and ***p ≤ 0.001). Cell viability and ROS generation assay.

The recombinant flHLF showed minimal inhibitory concentration at 50% (MIC_50_) of over 80 μM (Figure 2A), similar to the previous reports (Conneely, 2001; Samuelsen et al., 2004; Fedorka et al., 2018). Thus, it is likely that flHLF exhibits anti-inflammatory activities *via* direct interaction with the host cells and the inhibition of inflammation-causing pathogens (Drago-Serrano et al., 2017). Comparatively, rtHLF4 has an MIC_50_ of approximately 5 μM, an order of magnitude lower than flHLF (Figure 2B). Although rteHLF1 shows some form of anti-hemolytic properties, the protein could not achieve 50% inhibition despite using up to 100 μM of the protein (Figure 2C). rpHLF2’s MIC_50_ is above 50μM, showing a slight improvement over the flHLF (Figure 2D).

Our data indicate that all the purified proteins had anti-hemolytic properties, where rtHLF4 had the best activity. Although there is complete inhibition of the indomethacin-induced hemolytic activity, approximately 30% of hemolysis results from erythrocyte instability during the preparation process for the hemolytic assay. Hence, we followed the anti-hemolytic assay with an anti-proliferative assay and a reactive oxygen species (ROS) assay to investigate the protein toxicity and validate the inflammatory properties of these purified proteins.

We tested flHLF and various lactoferrin fragments’ cytotoxicity on non-malignant human colonic fibroblast cells and colorectal cancer cells using a cell viability assay. All the purified proteins do not inhibit the CCD-841-CON and CCD-18co fibroblast cell lines (Table 1, Supplementary Figure S3, S4) but inhibit HT29 colon cancer cells. rtHLF4, in particular, has a higher half-inhibitory concentration at 50% (IC_50_) in HT29, showing at least 50%–70% higher IC_50_ values than flHLF (Table 1). This observation suggests that the purified proteins have no cytotoxicity to normal healthy cells but presents anti-cancer properties. The IC_50_ values of flHLF, rteHLF1, and rpHLF2 tested against HT29 were below 120 μM (Table 1, Supplementary Figures S5A,C,D), whereas rtHLF4 has a lower IC_50_ value, below 10 μM after 3 and 24 h of treatment (Table 1, Supplementary Figure S5B). These results are consistent with the anti-cancer results from our earlier study (Pan et al., 2021).

**TABLE 1:** Cell viability and ROS generation of different colorectal cell lines treated with purified lactoferrin derivatives. Assays were conducted in triplicate.

	–	Cell viability (IC_50_)				ROS generation, treated vs. untreated (%)		
Cell lines	Lactoferrin fragment	3 h (IC_50_, μM)	IC_50_ fold change compared to flHLF	24 h (IC_50_, μM)	IC_50_ fold change compared to flHLF	10 μM	0.1 μM	0.05 μM
CCD-841-CON	FlHLF	149.1 ± 5.2	1.00	66.7 ± 3.3	1.00	89.2 ± 8.3	95.0 ± 3.8	111.0 ± 2.9
	rtHLF4	267.8 ± 8.8	1.80	60.4 ± 5.2	0.90	65.2 ± 4.3	98.5 ± 5.5	111.9 ± 1.8
	rteHLF1	149.4 ± 2.3	1.00	53.9 ± 1.1	0.81	83.2 ± 4.4	95.1 ± 2.0	106.6 ± 1.4
	rpHLF2	125.1 ± 3.4	0.84	67.3 ± 1.1	1.01	84.6 ± 6.7	97.2 ± 9.4	103.5 ± 2.7
CCD-18co	FlHLF	133.8 ± 3.3	1.00	71.7 ± 1.8	1.00	85.0 ± 2.3	94.8 ± 3.0	97.0 ± 4.2
	rtHLF4	186.2 ± 1.1	1.39	59.4 ± 1.2	0.83	61.3 ± 5.6	83.7 ± 0.5	94.5 ± 4.2
	rteHLF1	114.3 ± 2.0	0.85	53.1 ± 1.8	0.74	82.3 ± 1.8	95.7 ± 1.3	94.2 ± 5.4
	rpHLF2	104.2 ± 2.0	0.78	93.6 ± 1.3	1.31	74.2 ± 4.5	85.2 ± 6.2	96.1 ± 1.9
HT29	flHLF	83.5 ± 1.7	1.00	67.8 ± 1.4	1.00	87.0 ± 3.3	103.2 ± 11.1	117.7 ± 6.7
	rtHLF4	13.4 ± 2.1	0.16	3.6 ± 0.6	0.05	83.0 ± 5.1	80.8 ± 5.1	89.5 ± 5.5
	rteHLF1	100.5 ± 3.1	1.20	73.0 ± 1.6	1.08	104.7 ± 2.8	103.9 ± 3.1	110.7 ± 4.7
	rpHLF2	129.5 ± 2.6	1.55	40.9 ± 2.9	0.60	85.3 ± 7.2	89.0 ± 9.9	97.3 ± 8.6

The suppression of ROS generation by the purified proteins were used to validate its anti-inflammatory properties. ROS are considered prominent signaling molecules involved in the inflammatory disorder progression, often resulting from oxidative stress/injury. The tumor necrosis factor-α (TNF-α) triggers ROS production *via* the c-Jun N-terminal kinase activation. In this study, the purified proteins were added to fibroblast or cancer cells treated with TNF-α. rtHLF4 showed the highest inhibition on ROS generation in both the TNF-α-treated fibroblast cells, showing a decrease of over 40% of ROS generated when treated with 10 μM of the protein compared to the untreated fibroblast cells CCD-841-CON and CCD-18co (Table 1). Similarly, the other two proteins (rteHLF1 and rpHLF2) lowered the ROS generation by 10%–20%, where rteHLF1 showed a similar suppression profile to flHLF (Table 1). rtHLF4 also showed the highest suppression of ROS generation in HT29 cells when treated with 0.1 μM of the protein, further supporting the notion that rtHLF4 can prevent colorectal cancer pathogenesis (Table 1). Similarly, flHLF and rpHLF2 could suppress ROS generation to a lesser extent, whereas rteHLF1 showed no inhibition to the ROS generation (Table 1).

Lactoferrin can downregulate ROS levels by triggering the expression of superoxide dismutase (Thome et al., 1997), catalase (Nguyen et al., 2016), and glutathione peroxidase (Wei et al., 2004).

These lowered ROS levels generate a buffer against direct oxidative injuries imposed on the healthy colonic cells (Kruzel et al., 2017). On the other hand, ROS upregulation in cancer cells is attributed to improved anti-cancer activity, resulting in cancer cell apoptosis (Kuete et al., 2015; Zugic et al., 2016). The lactoferrin triggers the buildup of ROS levels within the cancer cell cytosol *via* the Caspase-8 activation, facilitating the cancer cell apoptosis (Ahmed et al., 2021). This observation concurs with the results of our study, where although the lactoferrin fragments can trigger ROS suppression in cancer cells, the suppression levels are two to three times lower than those found in the healthy fibroblast cells.

### Anti-Inflammatory Mechanism of Engineered Recombinant Lactoferrin

**FIGURE 3:**
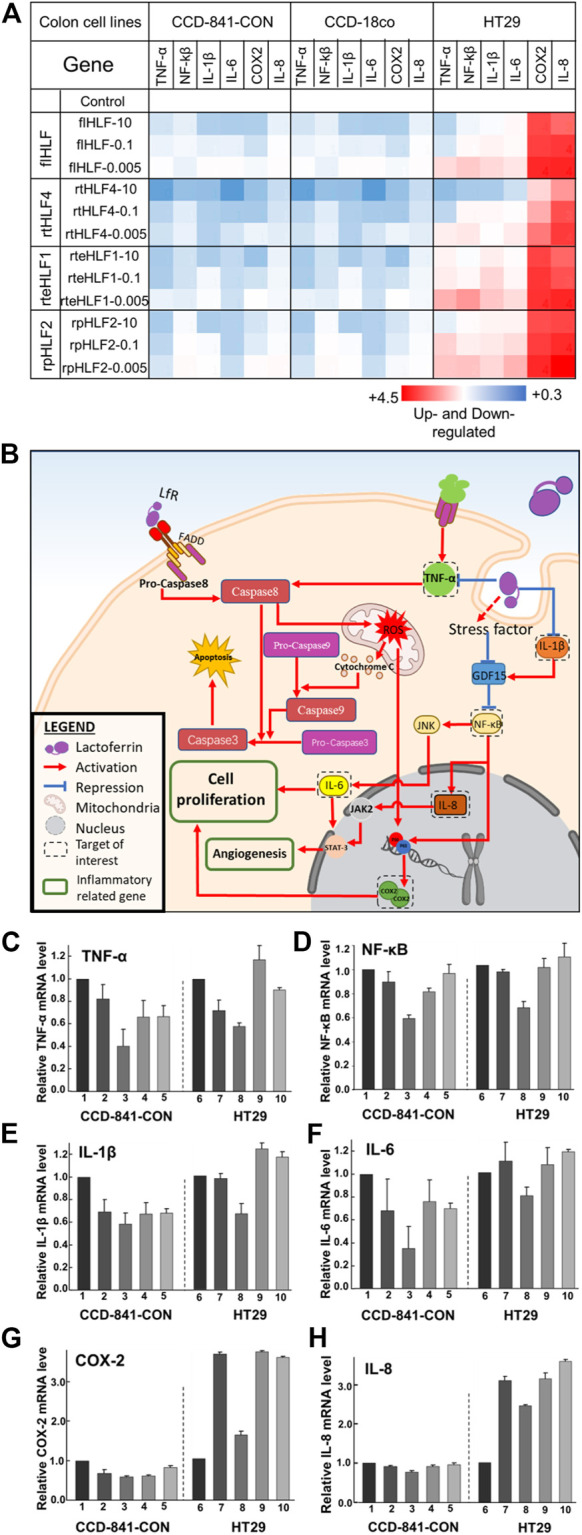
Anti-inflammatory mechanisms in human colon fibroblast cells and colorectal cancer cells following treatment with flHLF, rtHLF4, rteHLF1, and rpHLF2. **(A)** Quantitative PCR results of different genes in human colon fibroblast cells and colorectal cancer cells. Expression levels were compared against housekeeping gene ACTB (protein concentration: 10 μM/0.1 μM/0.005 μM). **(B)** The anti-inflammatory pathway in colon and cancer cells triggered by flHLF, rtHLF4, rteHLF1, and rpHLF2 treatment. Gene expression level in CCD-841-CON and HT29 cells **(C)** *TNF-α*; **(D)** *NF-κΒ*; **(E)** *IL-1β*; **(F)** *IL-6*; **(G)** *COX2*; **(H)** *IL-8* (Annotation for figures C-H: 1, TNFα-induced CCD-841-CON; 2, flHLF-10 μM; 3, rtHLF4-10 μM; 4, rteHLF1-10 μM; 5, rpHLF2-10 μM; 6, HT29 cells; 7, flHLF-10 μM; 8, rtHLF4-10 μM; 9, rteHLF1-10 μM; 10, rpHLF2-10 μM). Tests were conducted in triplicate.

In order to fully comprehend the regulatory genes governing the anti-inflammatory properties of purified proteins, we studied the transcriptomic levels of various biomarkers linked to the production of ROS and key inflammation-related genes (Figure 3A). We monitored TNF-α biomarker for its link to ROS production. The inflammation-related biomarkers are divided into the transcription factor NF-κΒ, pro-angiogenesis markers [(Interleukin-6 (IL-6) and Interleukin-8 (IL-8)], and cellular proliferation markers (IL-6 and COX2). We also investigated Interleukin-1β (IL-1β) levels as it regulates NF-κΒ levels. We further validated the cellular protein expression levels of these genes *via* Western blot (Supplementary Figures S6–S8). Based on the data, we mapped out the role of purified proteins in preventing inflammation (Figure 3B).

Both the colonic fibroblast cell lines treated with the purified proteins showed downregulated levels of TNF-α and the other inflammatory-related genes (Figures 3C–H, Supplementary Figures S6–S8). Of the four different proteins, rtHLF4 showed the best-conferred protection, while the other two proteins showed minimal difference compared to flHLF. Treatment of rtHLF4 on both the fibroblast cells showed approximately 50% lower protein levels of TNF-α (Figure 3C, Supplementary Figures S6A, S7A) and IL-6 (Figure 3F, Supplementary Figures S6D, S7D), 30% lower NF-κΒ (Figure 3D, Supplementary Figures S6B, S7B), and 10% lower IL-8 (Figure 3H, Supplementary Figures S6F, S7F) compared to flHLF treatment. Thus, generally, all lactoferrin variants could suppress ROS production and inhibit inflammation, with rtHLF4 showing the highest efficacy and potency.

Colorectal cancer cell line HT29 treated with flHLF and the various fragments also showed downregulation of ROS production and prevented inflammation *via* suppression of angiogenesis- and cellular proliferation-related genes (Figure 3B). Compared with flHLF, rtHLF4 treatment showed 20% lower IL-6 and IL-8 expression (Supplementary Figure S8). rtHLF4 also showed 20% lower NF-κΒ expression level (Figure 3D, Supplementary Figure S8B), 30% lower IL-Iβ (Figure 3E, Supplementary Figure S8C), and 60% lower COX-2 (Figure 3G, Supplementary Figure S8E) when compared against flHLF, outperforming the rteHLF1 and rpHLF2. Compared to the healthy fibroblast cells, the lesser suppression of ROS production is essential to facilitate the anti-cancer activity of rtHLF4, whereas the lowering of the IL-1β coupled to the GDF-15 upregulation significantly suppresses NF-κΒ activity (Figure 3B). This results in the inhibition of COX-2 production directly regulated by both NF-κΒ inactivation and the lowered ROS levels.

Earlier findings showed that TNF**-**α induces ROS production *via* caspase-8 activation, resulting in the mitochondrial outer membrane permeabilization (MOMP) (Roberge et al., 2014). The MOMP releases ROS and cytochrome C, activating caspase-9 (Kim and Park, 2003). Both caspases-8 and -9 activate caspase-3 resulting in cellular apoptosis (Elmore, 2007). The generated ROS species triggers p50/p65 complex, inducing COX2 expression (Narayanan et al., 2014) (Charalambous et al., 2009). COX-2 overexpression during inflammatory responses often results in the production of prostaglandins involved in colorectal cancer carcinogenesis (Tsatsanis et al., 2006). Our results are consistent with this observation, where lactoferrin suppresses TNF**-**α expression and prevents cancer carcinogenesis (Kim et al., 2012; Fedorka, 2017).

NF-κΒ activation is the key transcription factor controlling the pro-inflammatory genes, increasing the risk of colitis-associated and other forms of gastrointestinal cancer (Popivanova et al., 2008). NF-κΒ also induces IL-6 through JNK (Lin et al., 2011), playing a role in cancer cell proliferation, stimulating tumor growth and the proliferation of premalignant enterocytes (Becker et al., 2005). Suppression of NF-κΒ *via* IL-1β (an acute pro-inflammatory cytokine) (Xue et al., 2015) or through GDF-15 ameliorates the inflammation within the host. Our earlier study showed that lactoferrin upregulates GDF15 expression, thereby inhibiting NF-κΒ (Pan et al., 2021). NF-κΒ activation also results in COX-2 upregulation as observed in colonic adenoma and carcinoma (Rehemtulla, 2013) (Charalambous et al., 2009) (Hai et al., 2016). NF-κΒ activation also upregulates IL-8, promoting angiogenesis *via* JAK2 and STAT3 (Fu et al., 2015; Di et al., 2020). Our results suggest that all the lactoferrin variants, with rtHLF4 in particular, can downregulate NF-κΒ, thus showing anti-inflammation properties and inhibiting tumor growth.

## Conclusion

In summary, the flHLF and the lactoferrin variants (rtHLF4, rteHLF1, and rpHLF2) show anti-inflammatory and anti-oxidation activities by inhibiting TNF-α induced generation of ROS in human non-malignant colonic fibroblasts cells. These proteins also exhibited no cytotoxicity to the fibroblast cells. However, in cancer cells, rteHLF1 and rpHLF2 do not influence ROS production but inhibit the expression of pro-inflammatory factors. Although these proteins were expressed recombinantly in an *E. coli* host, lacking the post-translational modifications of the protein, these lactoferrin fragments can exert its bioactivity, indicating that the bioactivity is independent to its glycosylation status. rtHLF4 showed the most promise, exhibiting higher bioactivity compared to the flHLF, providing a viable alternative for IBD treatment and prevention in the future.

## Data Availability

The original contributions presented in the study are included in the article/Supplementary Material; further inquiries can be directed to the corresponding authors.

## Abbreviations

ACTB, beta actin; COX-2, cyclo-oxygenase-2; flHLF, full-length lactoferrin; IL-1β, interleukin 1 beta; IL-6, interleukin 6; IL-8, interleukin 8; NF-κB, nuclear factor kappa B; rpHLF2, recombinant engineered-lactoferrin 2; rteHLF1, recombinant engineered-lactoferrin 1; rtHLF4, recombinant engineered-lactoferrin 4; SDS-PAGE, sodium dodecyl sulfate-polyacrylamide gel electrophoresis; TNF-α, tumor necrosis factor-alpha.
