# Supplementary material for: Evaluation of the Anti-Inflammatory and Anti-Oxidative Effects of Therapeutic Human Lactoferrin Fragments
Source: Front Bioeng Biotechnol. 2021 Nov 30;9:779018. doi: 10.3389/fbioe.2021.779018 (PMC8671034; doi:10.3389/fbioe.2021.779018)
Supplement: Supplementary file 1 [file DataSheet1.docx]

Supplementary Material

Evaluation of the anti-inflammatory and anti-oxidative effects of therapeutic human lactoferrin fragments

Yu Pan^1†^, Zhao Liu^1†^, Yijie Wang ^1^, Linshen Zhang^1^, Niying Chua^2^, Jun Chen^1*^, and Chun Loong Ho^1*^

† These authors have contributed equally to this work and share first authorship

^1^Department of Biomedical Engineering, Southern University of Science and Technology (SUSTech), Shenzhen, China.

^2^School of Biological Sciences, Nanyang Technological University, Singapore.

*** *Correspondence:***Ho Chun Loong

E-mail: [hejl@sustech.edu.cn](mailto:hejl@sustech.edu.cn)

Chen Jun

E-mail: [Chenj3@sustech.edu.cn](mailto:Chenj3@sustech.edu.cn)
Department of Biomedical Engineering, Southern University of Science and Technology (SUSTech), Shenzhen 518005, China.

| **No** | **Title** | **Page** |
| --- | --- | --- |
| 1 | Table. S1 Real-time reverse transcription-polymerase chain reaction (RT-PCR) primer sequence. | 3 |
| 2 | Table. S2 Fragment of rtHLF4, rteHLF1 and rpHLF2 primer sequence | 4 |
| 3 | Fig. S1 Sequence of flHLF, rtHLF4, rteHLF1 and rpHLF2 (flHLF: 1~692; rtHLF4: 73~535; rteHLF1: 73~312; rpHLF2: 8~211; iron-binding site: 61,122,118,186,247,93 (indicated as blue color, italic); N-glycosylation sites:138,479,624 (indicated as red color, italic)). | 5 |
| 4 | Fig S2 Percentage of LPS in full-length lactoferrin and various lactoferrin fragments. (Positive control: UTI89) | 6 |
| 5 | Fig S3 Cell proliferation assay of four lactoferrin proteins in CCD-841-CON human colonic epithelial cell. | 7 |
| 6 | Figure S4 Cell proliferation assay of four lactoferrin proteins in CCD-18co human colonic epithelial cell. | 8 |
| 7 | Figure S5 Cell proliferation assay of four lactoferrin proteins in HT29 human colorectal adenocarcinoma cell. | 9 |
| 8 | Figure S6 Full length lactoferrin, rtHLF4, rteHLF1 and rpHLF2 with different concentration induce (a) *TNF-α*, (b) *NF-κΒ*, (c) *IL-1β*, (d) *IL-6*, (e) *COX-2*, (f) *IL-8* gene expression and Western blot in human colonic epithelial CCD-841-CON cell. | 14 |
| 9 | Figure S7 Full length lactoferrin, rtHLF4, rteHLF1 and rpHLF2 with different concentration induce (a) *TNF-α*, (b) *NF-κΒ*, (c) *IL-1β*, (d) *IL-6*, (e) *COX-2*, (f) *IL-8* gene expression and Western blot in human colonic epithelial CCD-18co cell | 17 |
| 10 | Figure S8 Full length lactoferrin, rtHLF4, rteHLF1 and rpHLF2 with different concentration induce (a) *TNF-α*, (b) *NF-κΒ*, (c) *IL-1β*, (d) *IL-6*, (e) *COX-2*, (f) *IL-8* gene expression and Western blot in HT29 human colorectal adenocarcinoma cells. | 18 |

**Table S1: Real-time reverse transcription-polymerase chain reaction (RT-PCR) primer sequence**

| **Gene** |  | **Primer sequence** | **Product length** |
| --- | --- | --- | --- |
| ACTB | sense (5’-3’) | GGACTTCGAGCAAGAGATGG | 234bp |
|  | antisense (5’-3’) | AGCACTGTGTTGGCGTACAG |  |
| NF-κB | sense (5’-3’) | CACTGTAACTGCTGGACCCAAGG | 86bp |
|  | antisense (5’-3’) | CGCCTCTGTCATTCGTGCTTCC |  |
| TNF-α | sense (5’-3’) | AAGGACACCATGAGCACTGAAAGC | 82bp |
|  | antisense (5’-3’) | AGGAAGGAGAAGAGGCTGAGGAAC |  |
| IL-1β | sense (5’-3’) | GGACAGGATATGGAGCAACAAGTGG | 121bp |
|  | antisense (5’-3’) | TCATCTTTCAACACGCAGGACAGG |  |
| IL-6 | sense (5’-3’) | GACAGCCACTCACCTCTTCAGAAC | 131bp |
|  | antisense (5’-3’) | GCCTCTTTGCTGCTTTCACACATG |  |
| IL-8 | sense (5’-3’) | AACTTTCAGAGACAGCAGAGCACAC | 125bp |
|  | antisense (5’-3’) | CACACAGTGAGATGGTTCCTTCCG |  |
| COX-2 | sense (5’-3’) | GGGTTGCTGGTGGTAGGAATGTTC | 112bp |
|  | antisense (5’-3’) | CTGGTATTTCATCTGCCTGCTCTGG |  |

**Table S2: Fragment of rtHLF4, rteHLF1 and rpHLF2 primer sequence**

| **Fragment name** | |  | **Primer sequence** | **Enzymes sit in Vector** |
| --- | --- | --- | --- | --- |
| rtHLF4 | rtHLF4_Fwd | | 5'- CCCTCGAGAAGTTAAGACCAGTTGCTGC -'3 | NheI, XhoI |
|  | rtHLF4_Rev | | 5'- CGGCTAGCCATCTGAAAGCACCAGTGTA -'3 |  |
| rteHLF1 | rteHLF1_Fwd | | 5'-CGGGATCCTACAAGTTAAGACCAGTTG -'3 | BamHI, XhoI |
|  | rteHLF1_Rev | | 5'-CCCTCGAGTGGAACTCTAGAAAAACCG -'3 |  |
| rpHLF2 | rpHLF2_Fwd | | 5'-CGGGATCCCAATGGTGTGCTGTT -'3 | BamHI, XhoI |
|  | rpHLF2_Rev | | 5'-CCCTCGAGTCTAATGAAAGCAACATC -'3 |  |


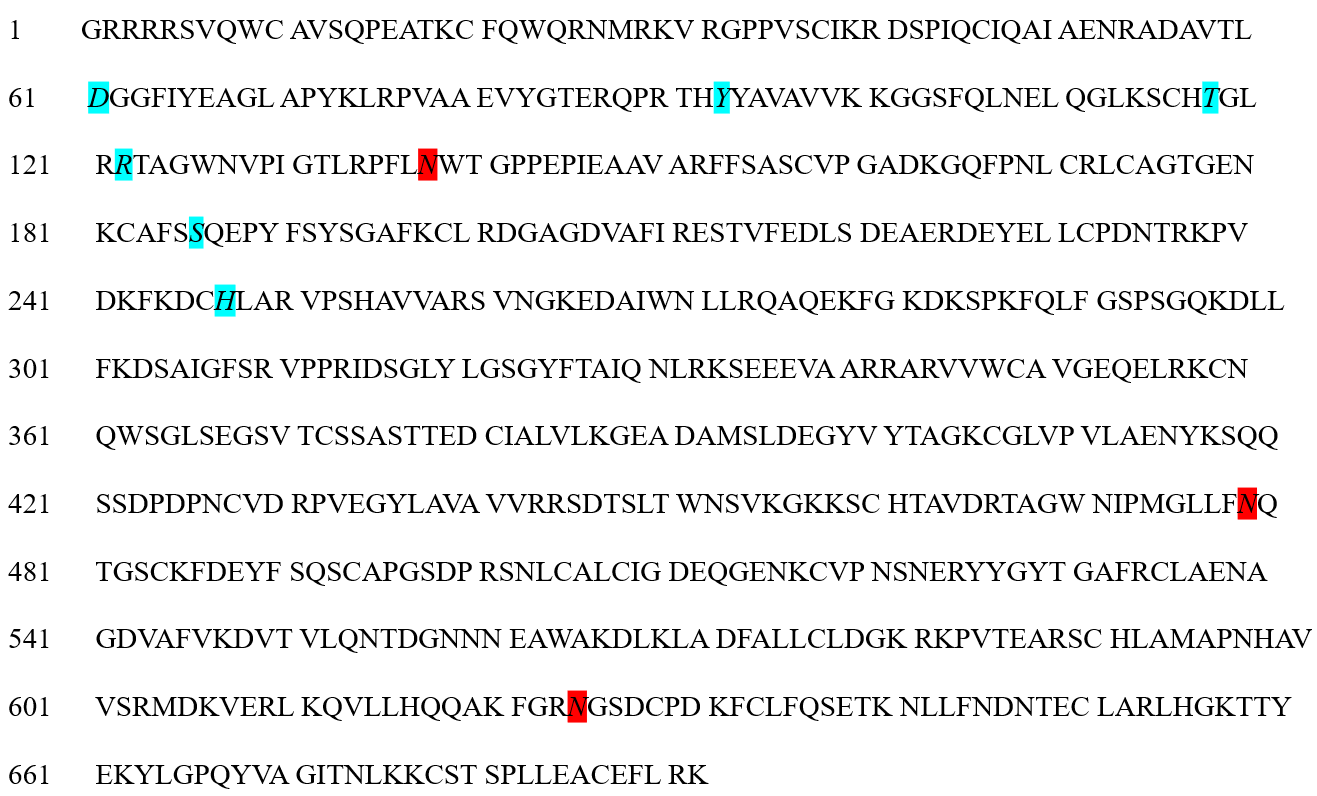


[Fig. S1 Sequence of flHLF, rtHLF4](#_Toc69823731), rteHLF1 and rpHLF2 (flHLF: 1~692; rtHLF4: 73~535; rteHLF1: 73~312; rpHLF2: 8~211; iron-binding site: 61,122,118,186,247,93 (indicated as blue background, italic); N-glycosylation sites:138,479,624 (indicated as red background, italic)).


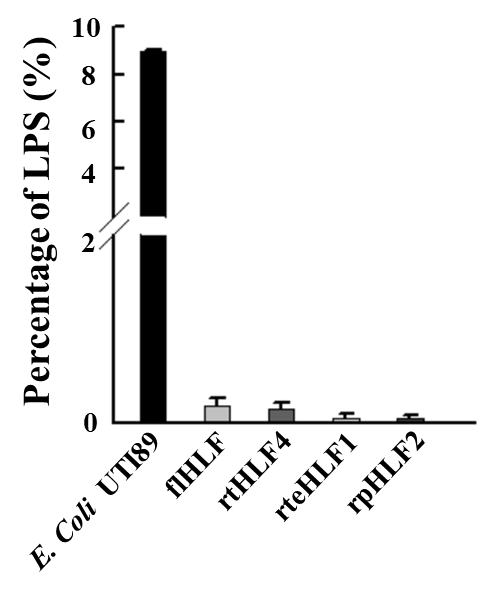


Figure S2: Percentage of LPS in full-length lactoferrin and various lactoferrin fragments. (Positive control: E. coli UTI89, percentage of LPS in flHLF and rtHLF4: ~0.02%, percentage of LPS in flHLF and rtHLF4: ~0.01%,)


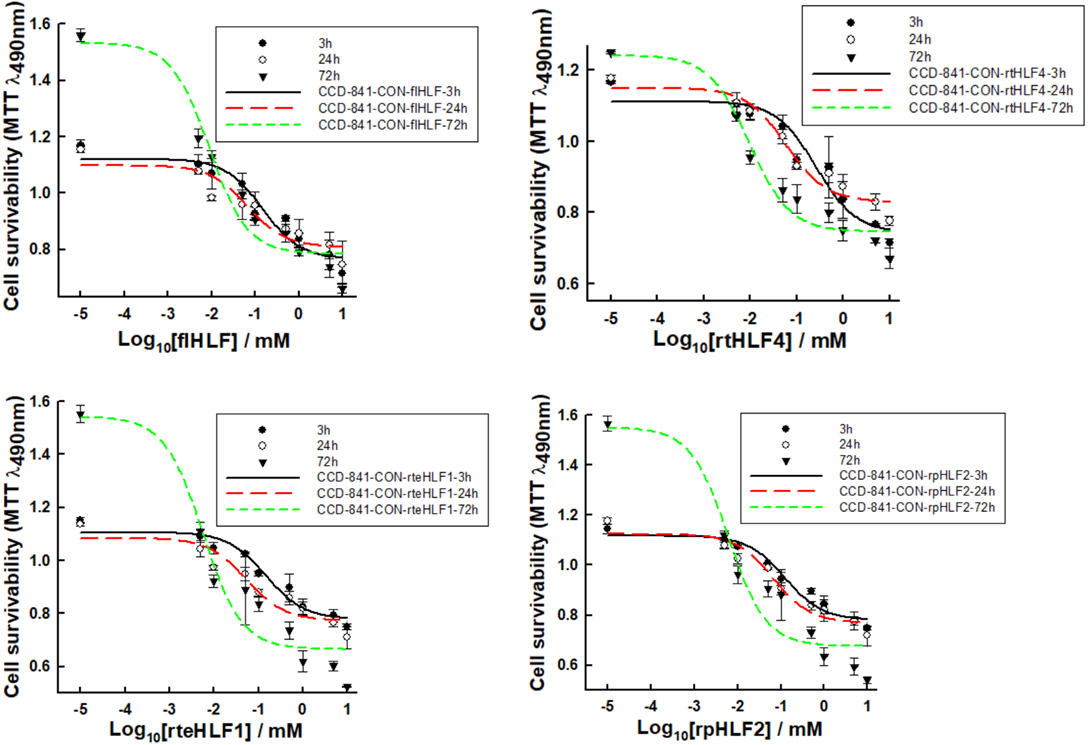


(d)

(a)

(c)

(b)

Fig S2: Cell proliferation assay of four lactoferrin proteins in CCD-841-CON human colonic epithelial cell. Dose-response of CCD-841-CON against varying concentration of (a) flHLF; (b) rtHLF4; (c) rteHLF1 and (d) rpHLF2.


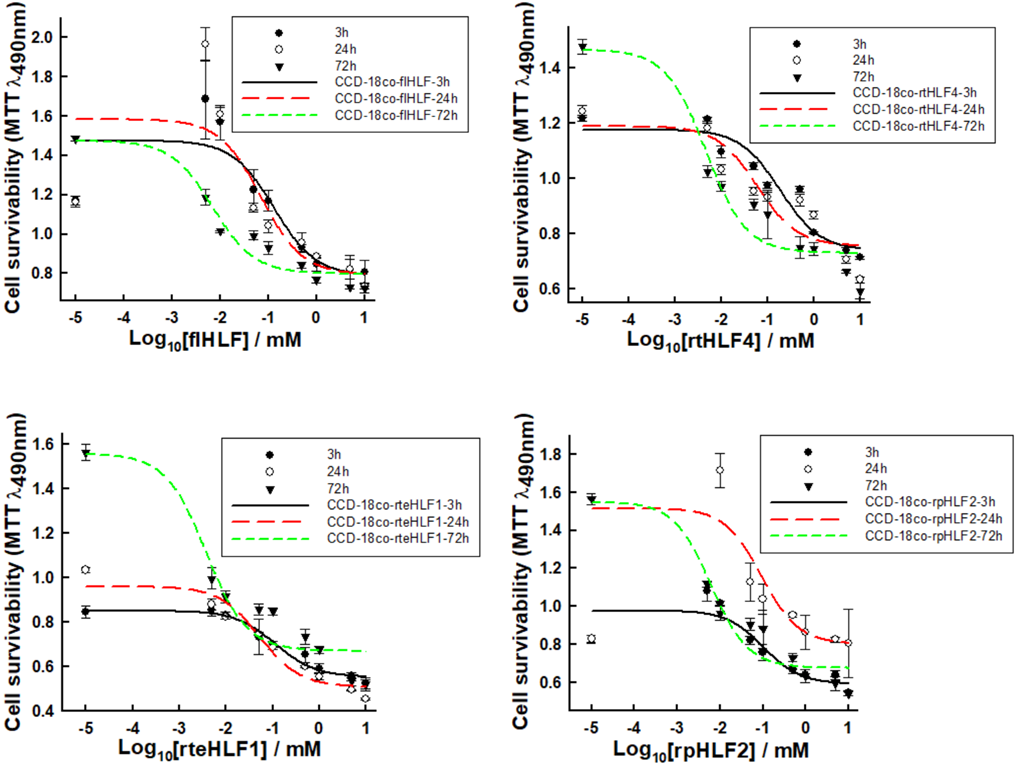


(d)

(c)

(b)

(a)

Fig S3: Cell proliferation assay of four lactoferrin proteins in CCD-18co human colonic epithelial cell. Dose-response of CCD-18co against varying concentration of (a) flHLF; (b) rtHLF4; (c) rteHLF1 and (d) rpHLF2.

(b)

(a)


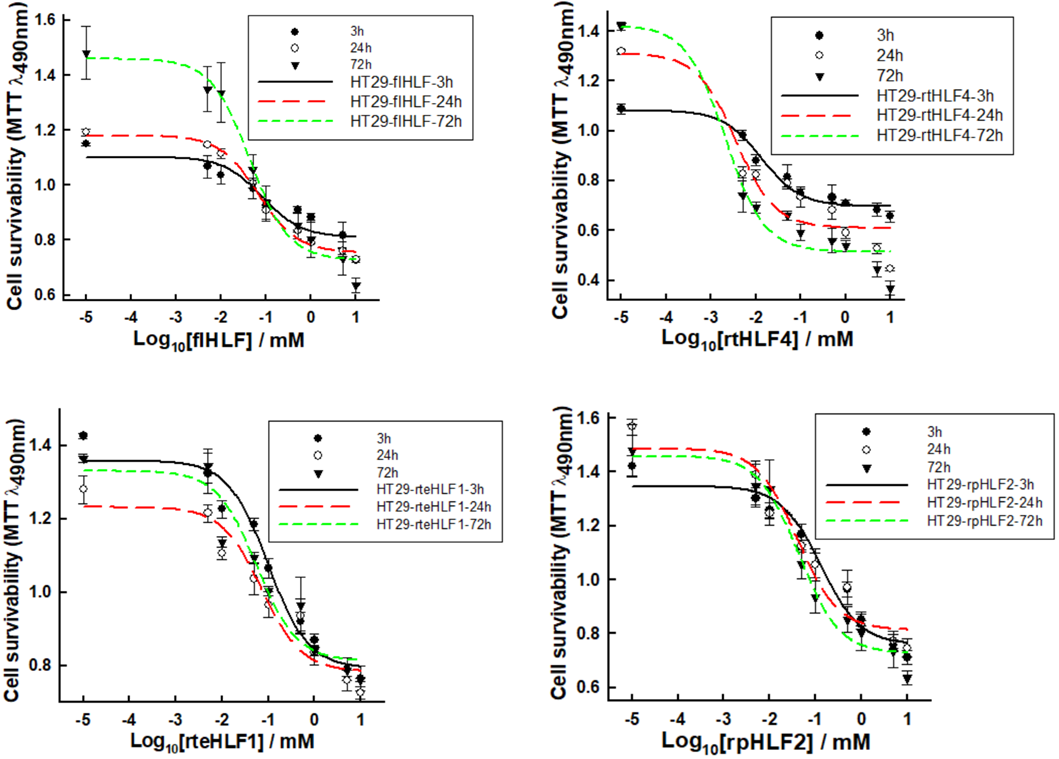


(d)

(c)

Fig S4: Cell proliferation assay of four lactoferrin proteins in HT29 human colorectal adenocarcinoma cells. Dose-response of HT29 against varying concentration of (a) flHLF; (b) rtHLF4; (c) rteHLF1 and (d) rpHLF2.


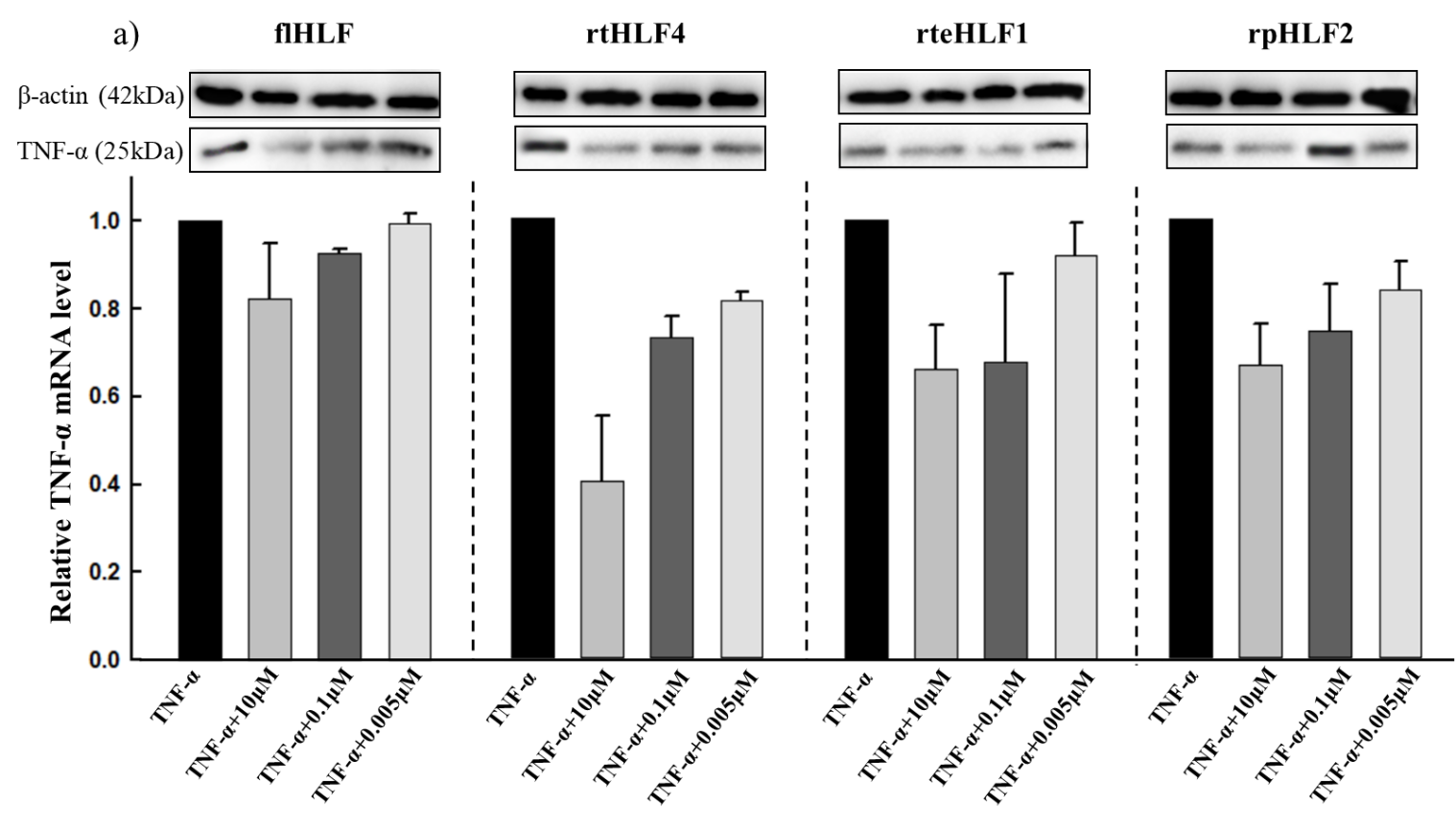


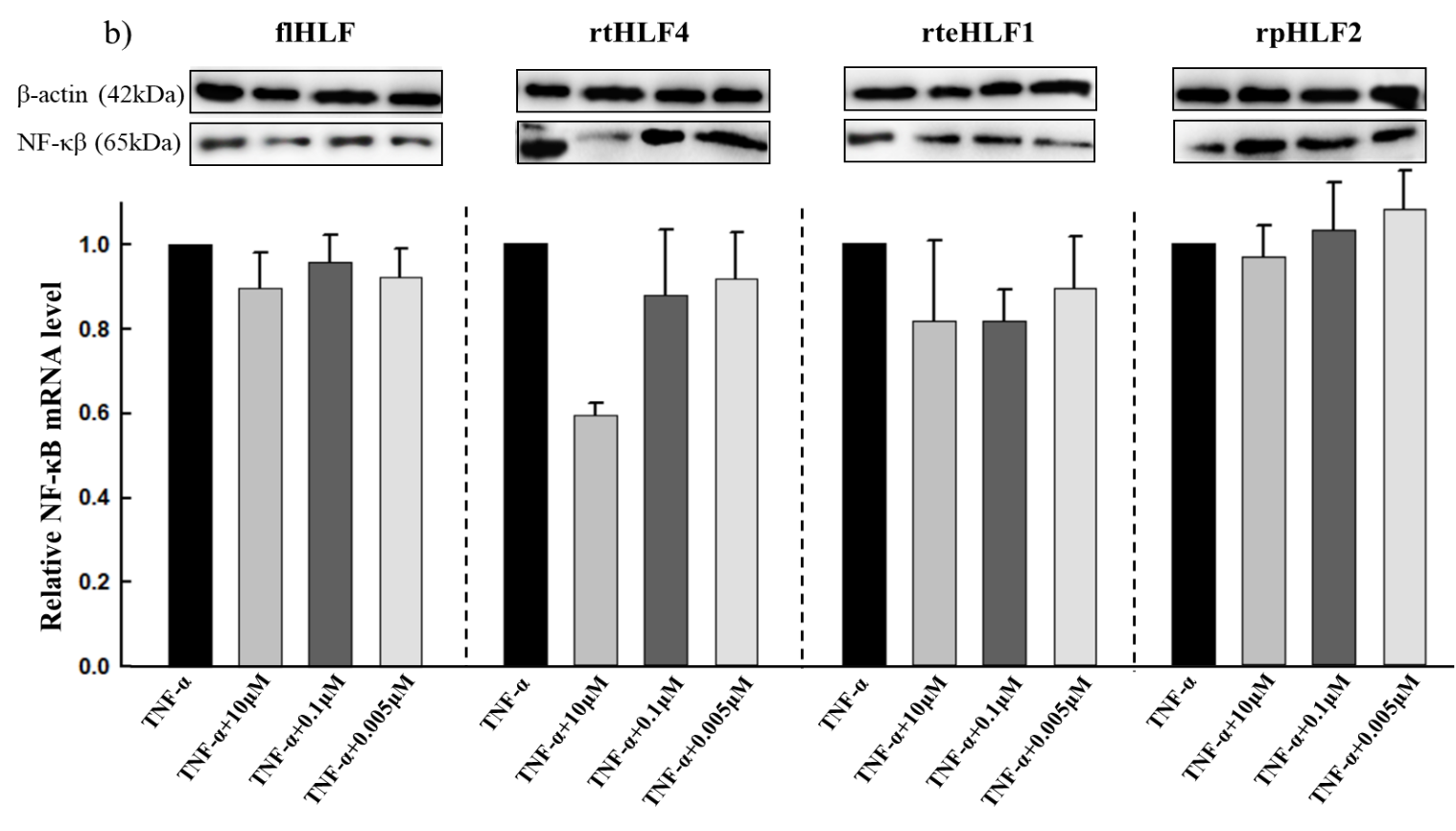


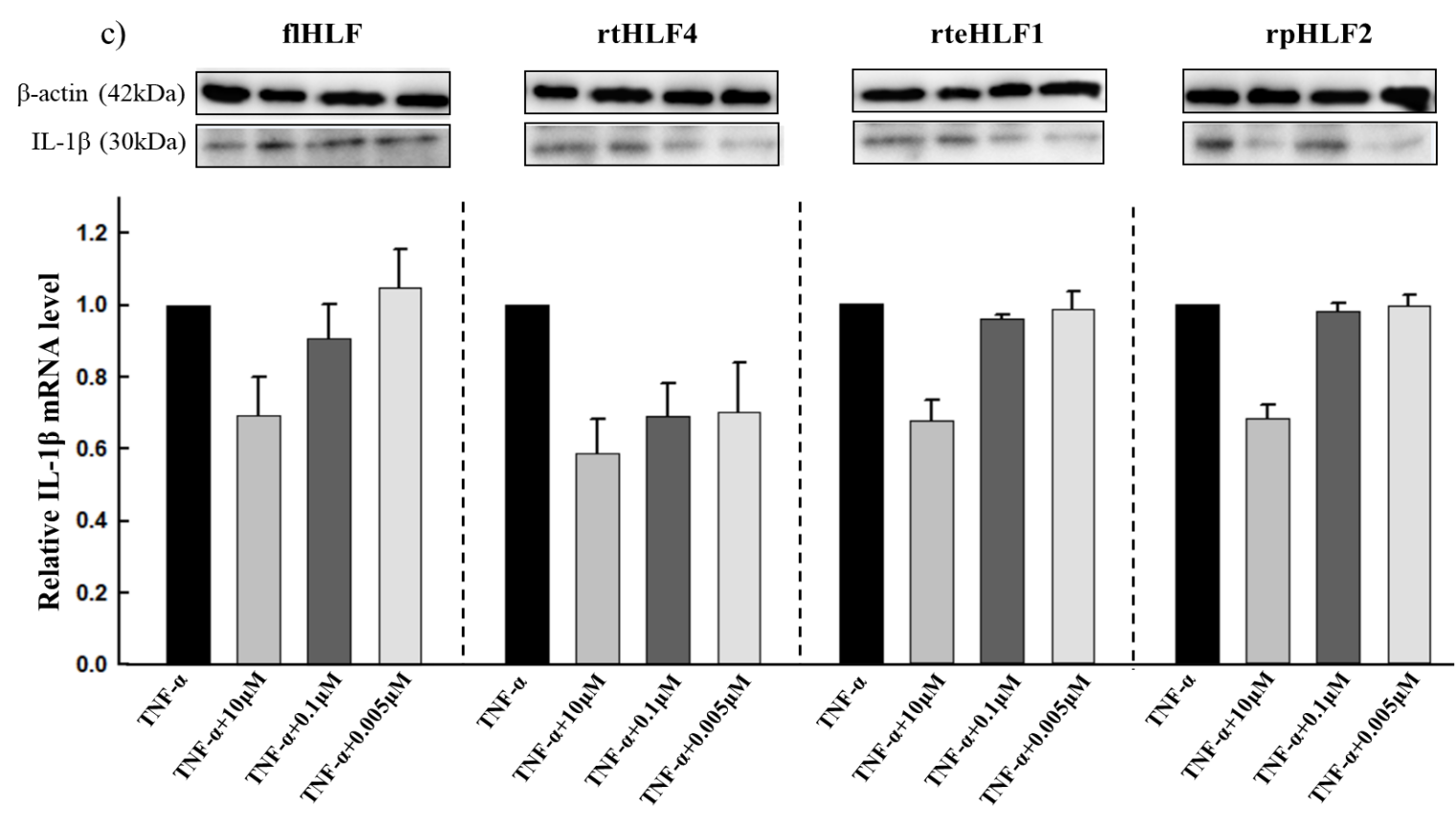


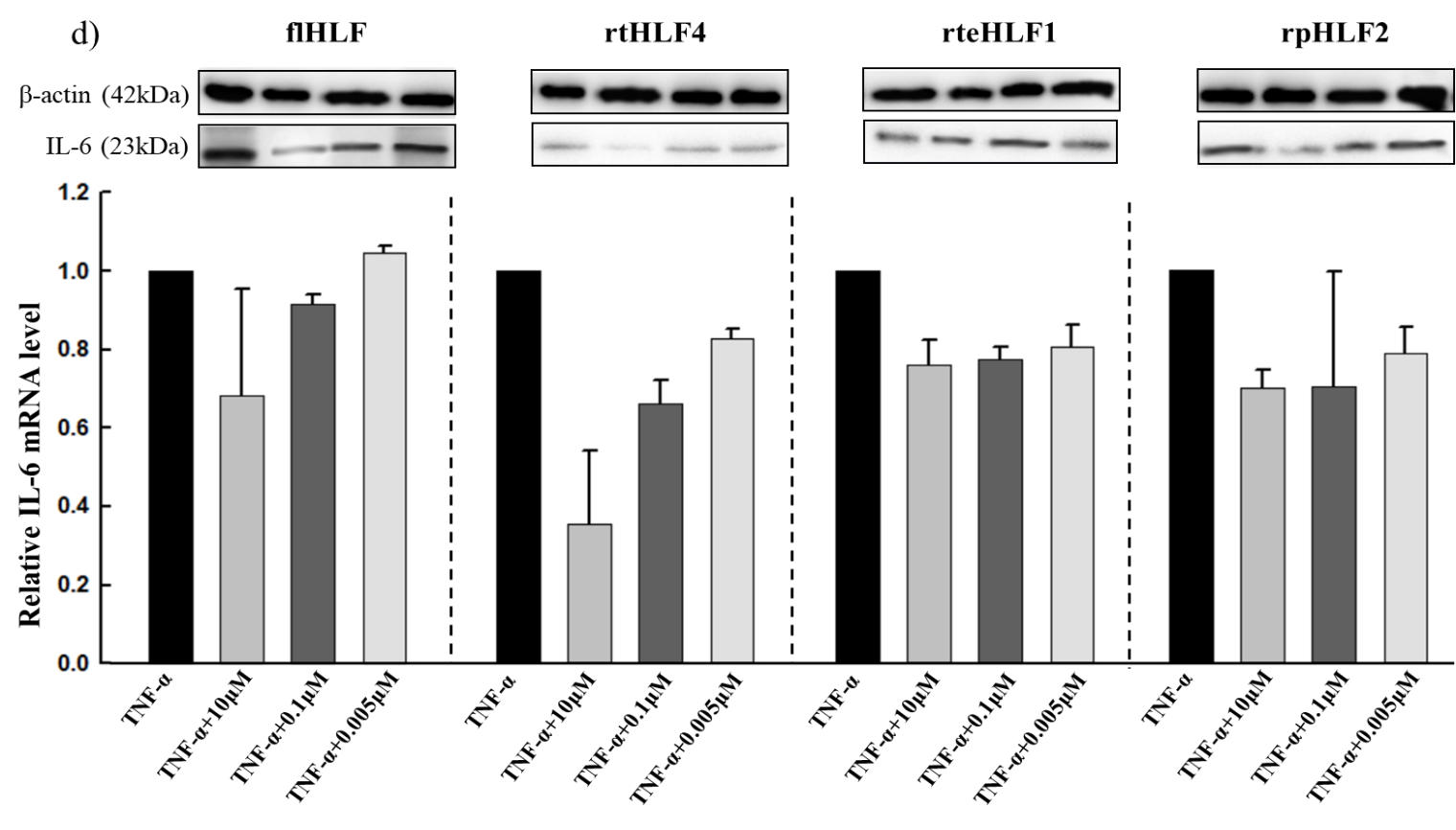


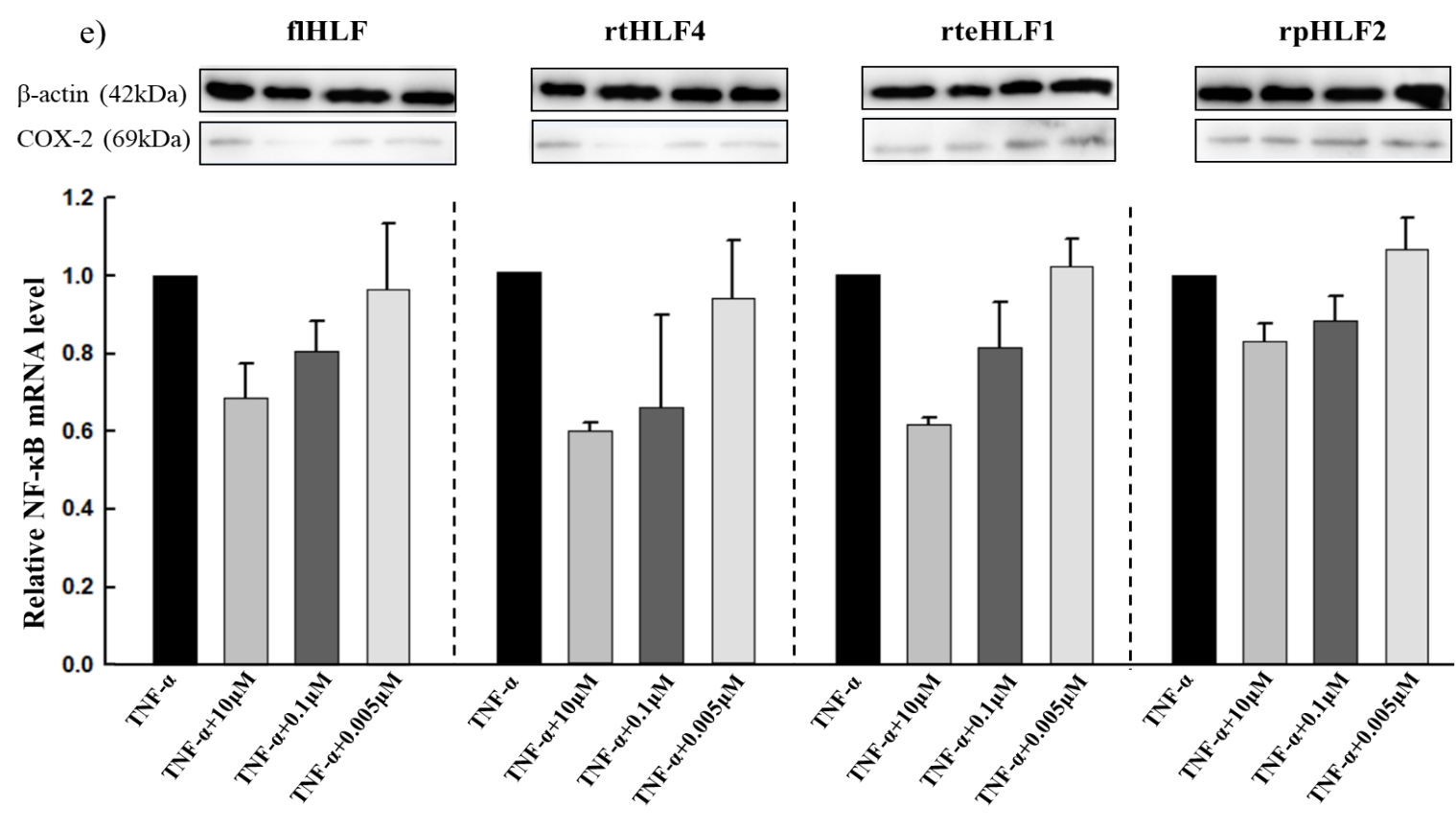


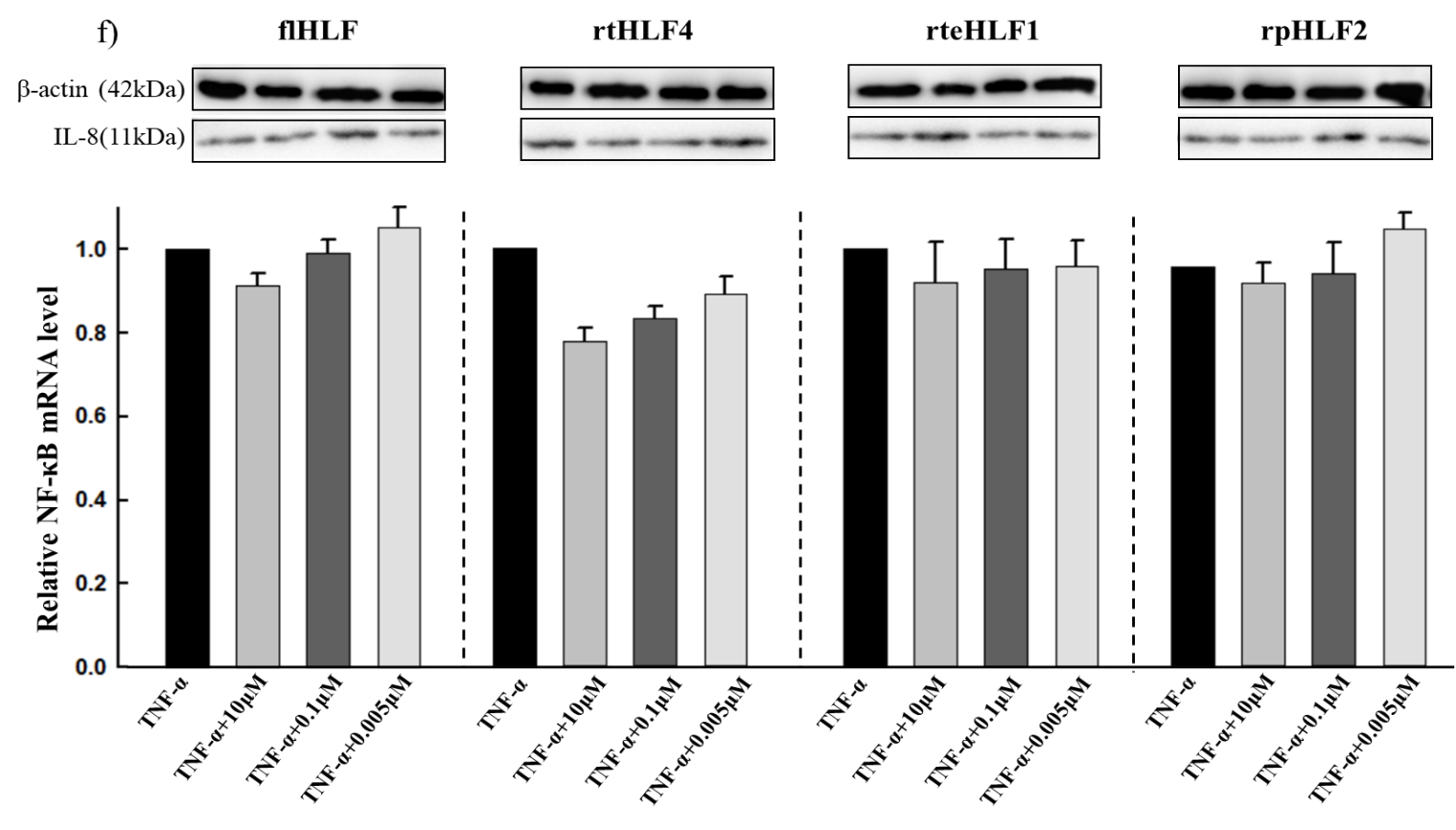


Fig S5: Full length lactoferrin, rtHLF4, rteHLF1 and rpHLF2 with different concentration induce (a) *TNF-α*, (b) *NF-κΒ*, (c) *IL-1β*, (d) *IL-6*, (e) *COX-2*, (f) *IL-8* gene expression and Western blot in human colon epithelial CCD-841-CON cells.


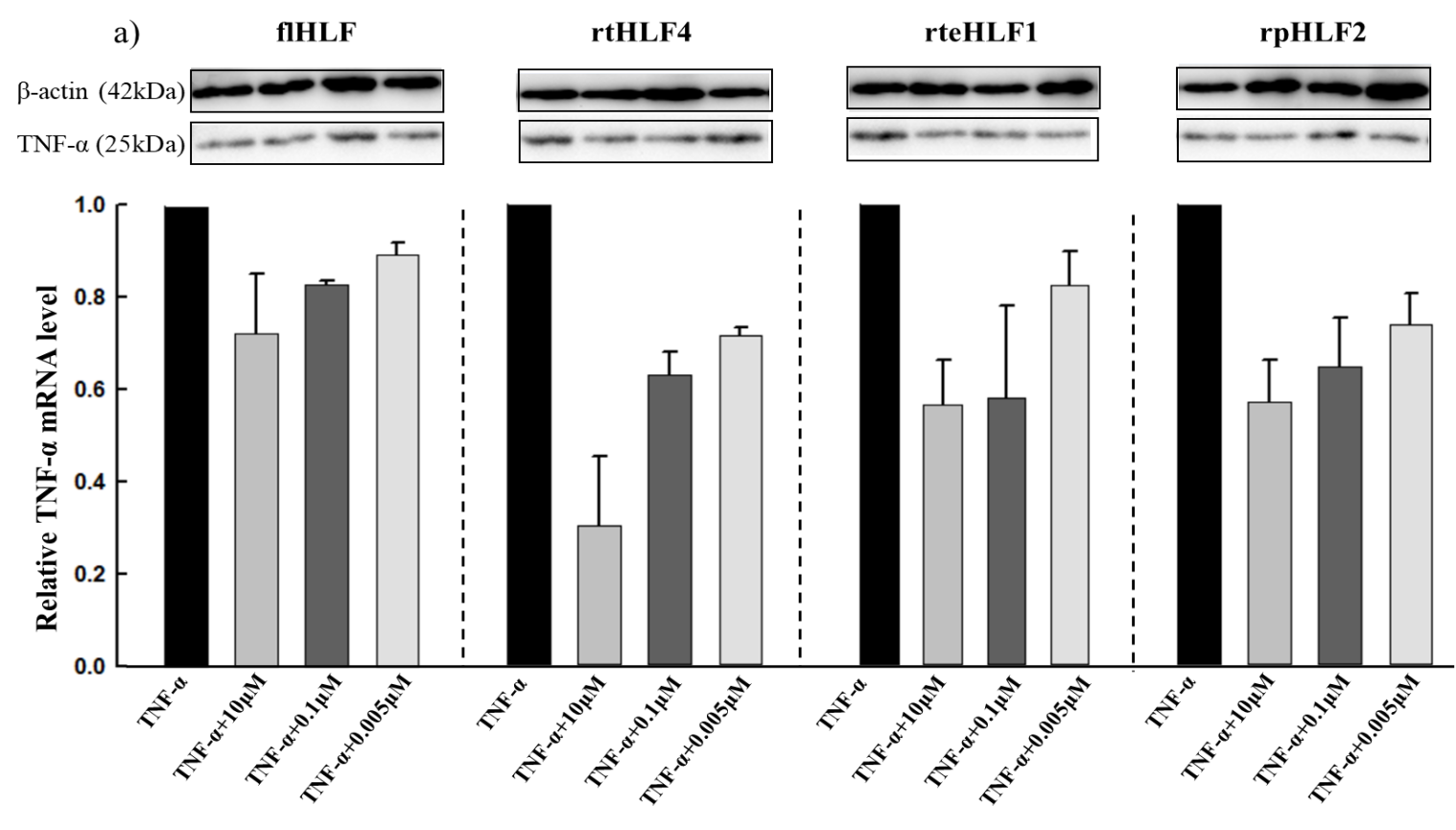


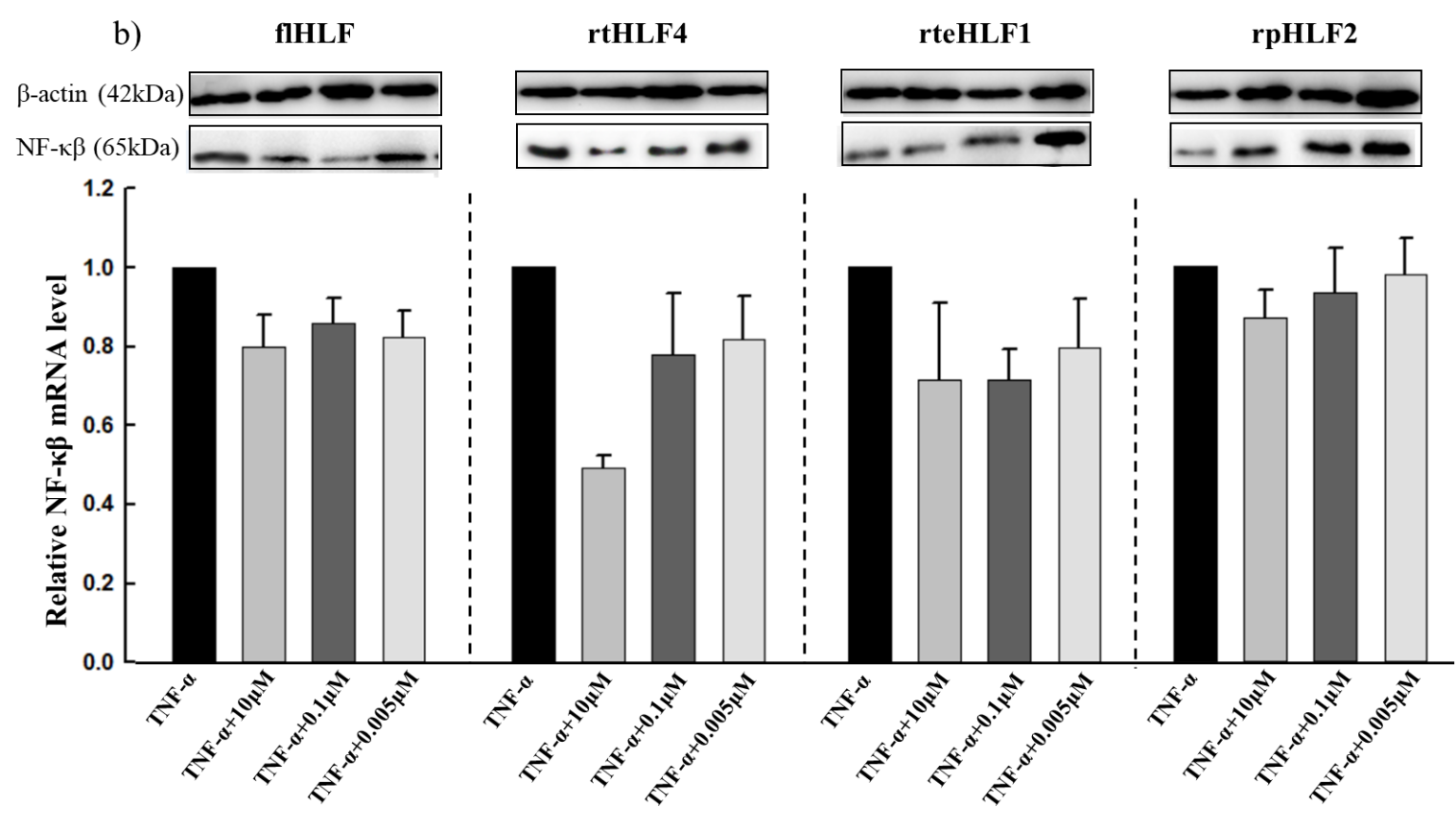


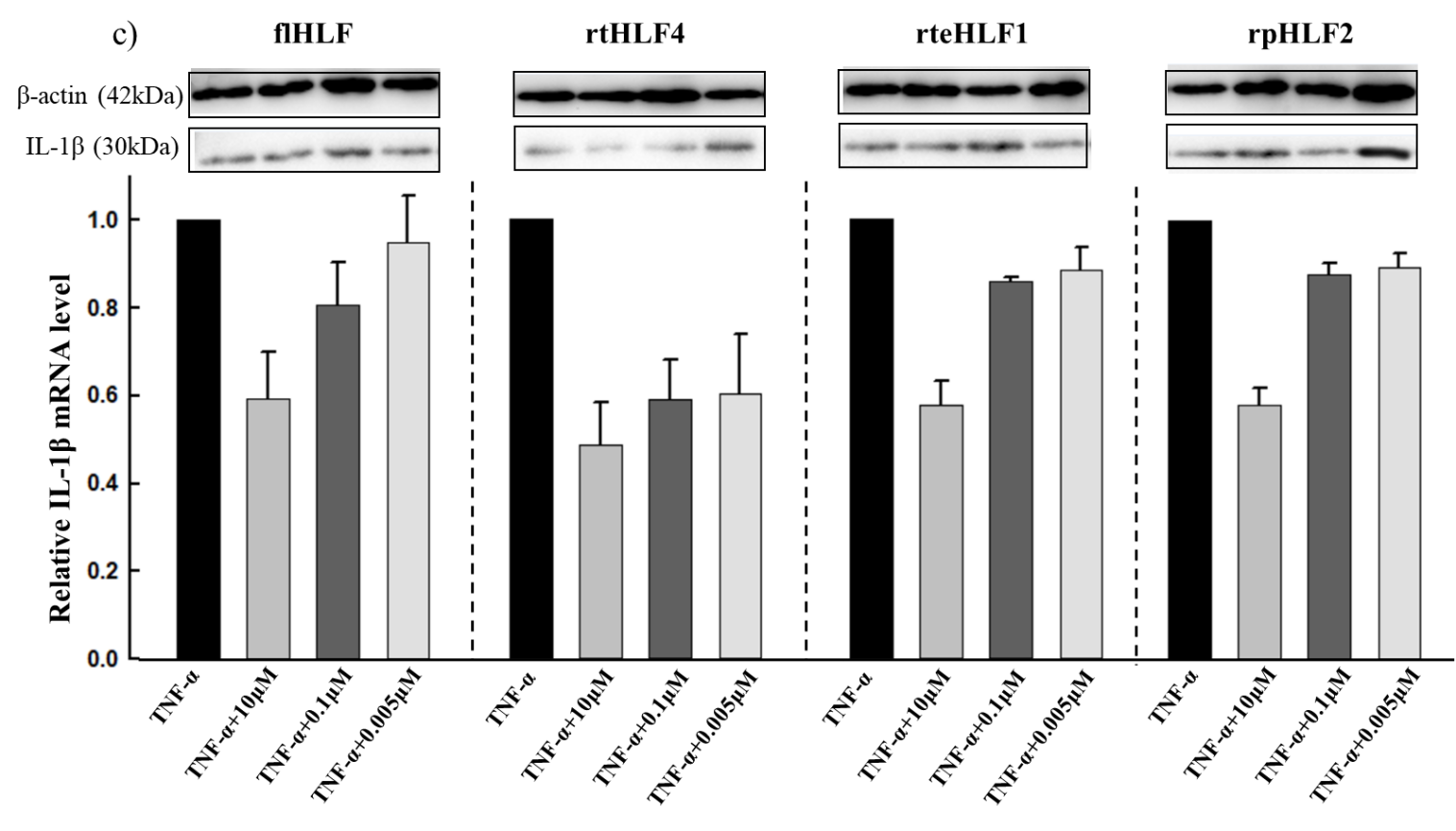


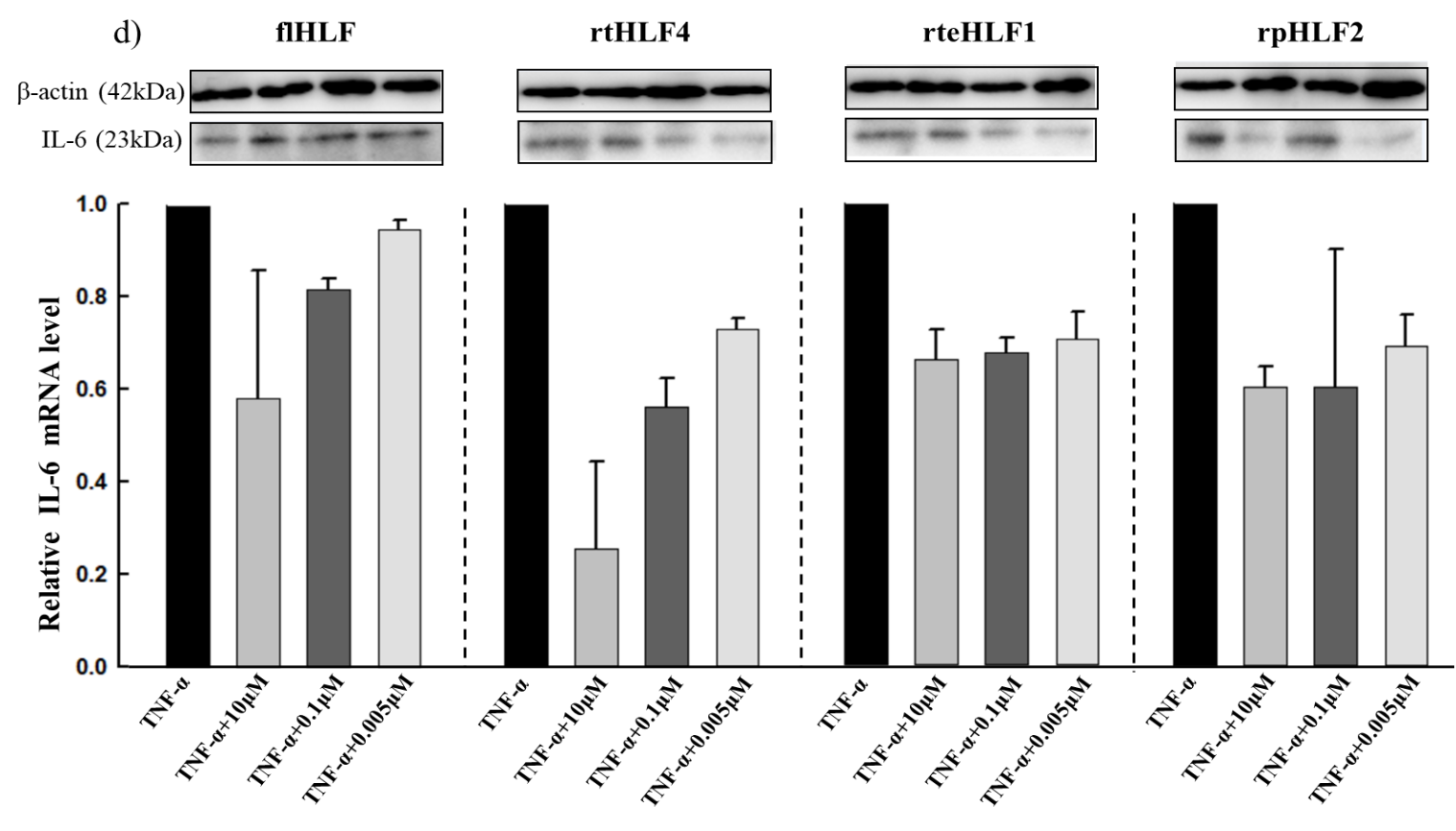


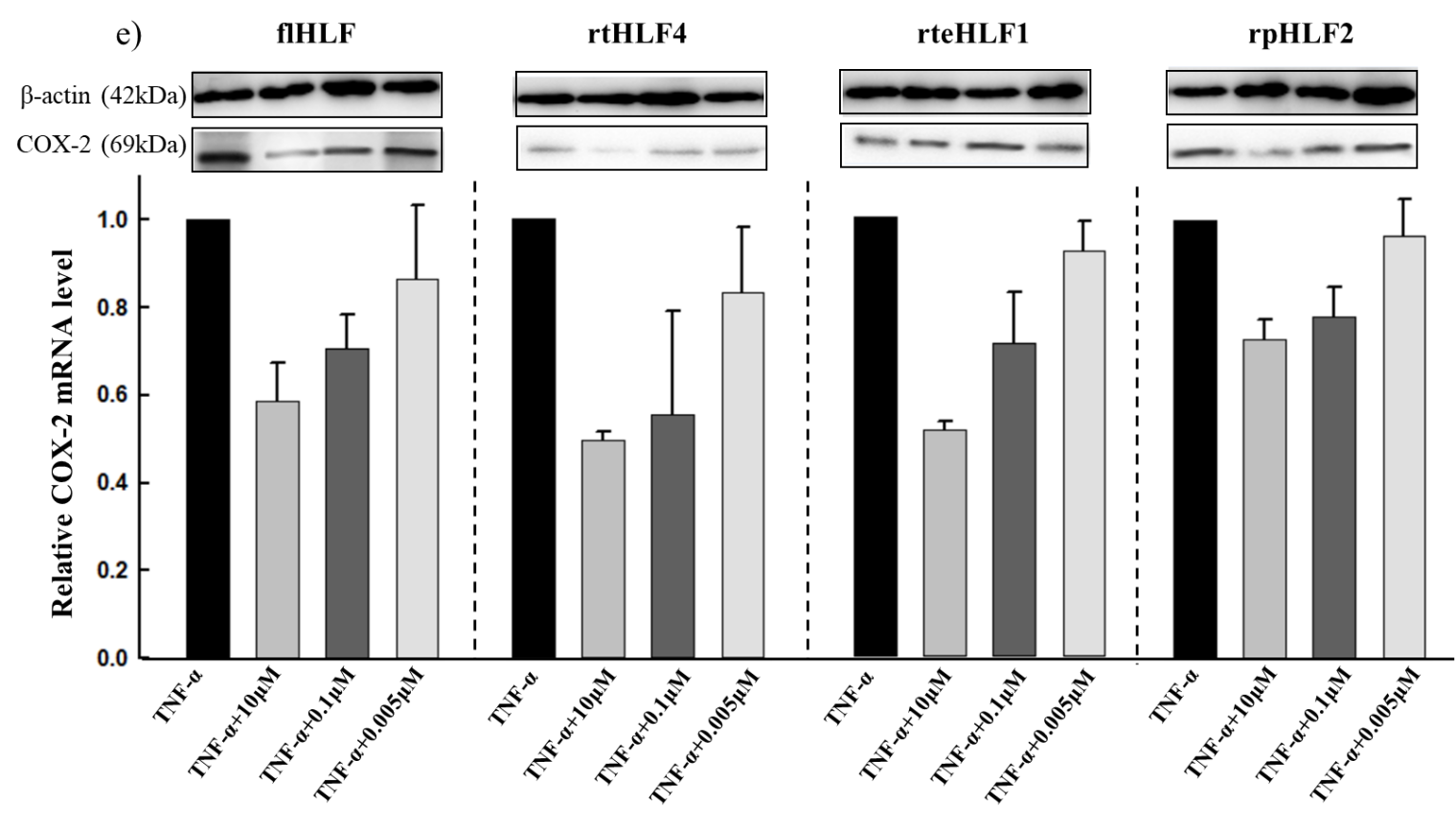


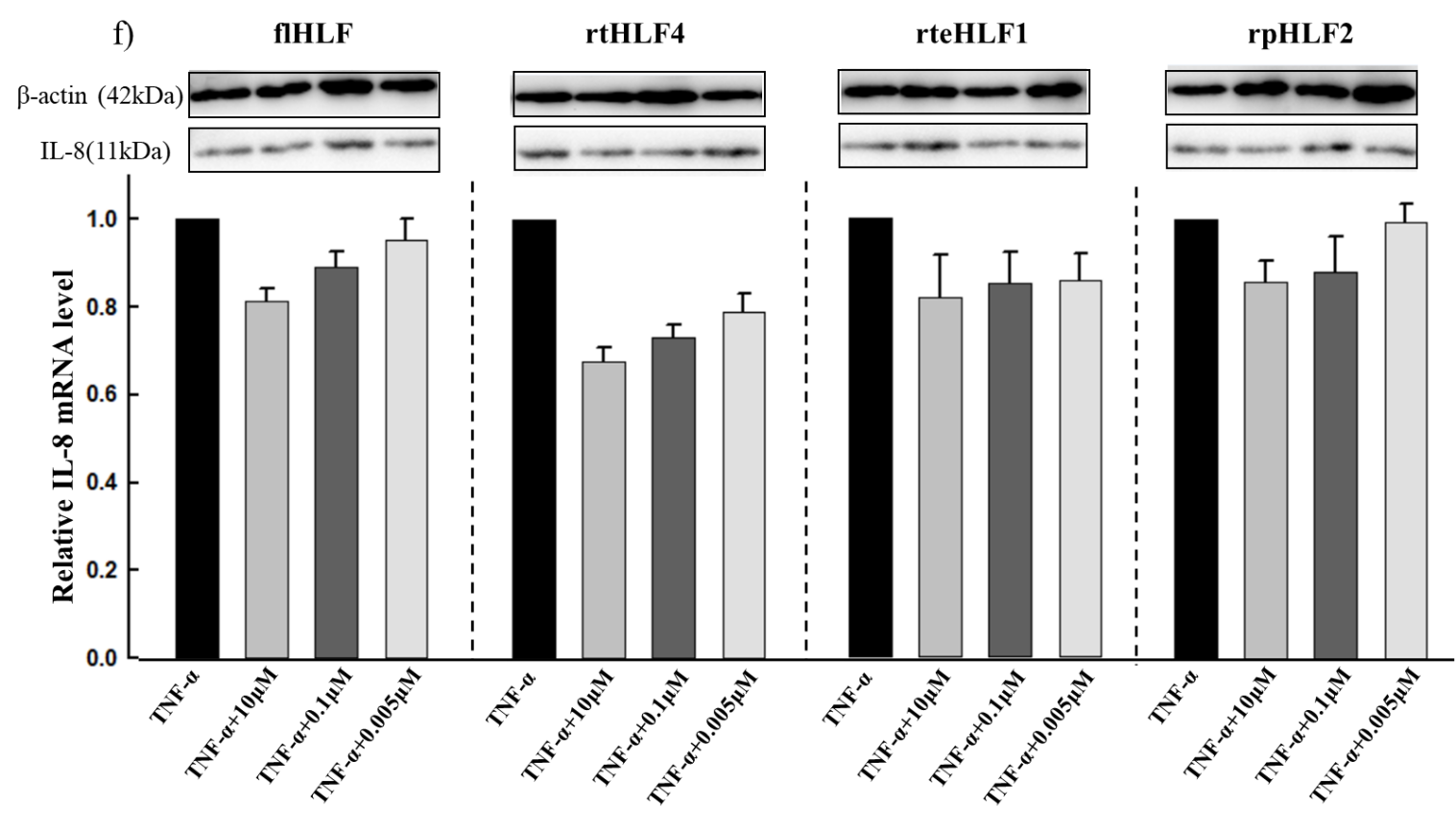


Fig S6: Full length lactoferrin, rtHLF4, rteHLF1 and rpHLF2 with different concentration induce (a) *TNF-α*, (b) *NF-κΒ*, (c) *IL-1β*, (d) *IL-6*, (e) *COX-2*, (f) *IL-8* gene expression and Western blot in human colon epithelial CCD-18co cells.


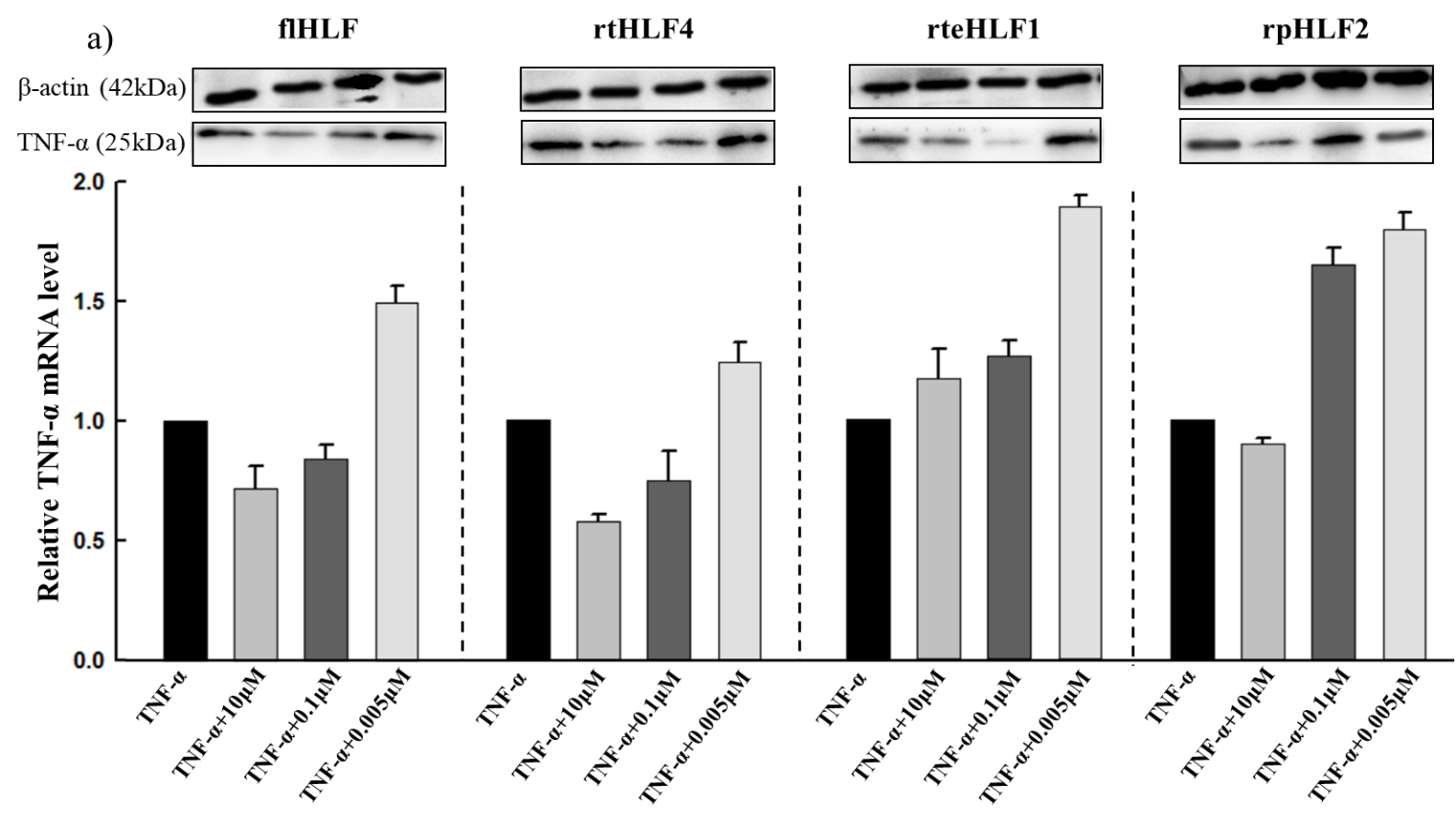


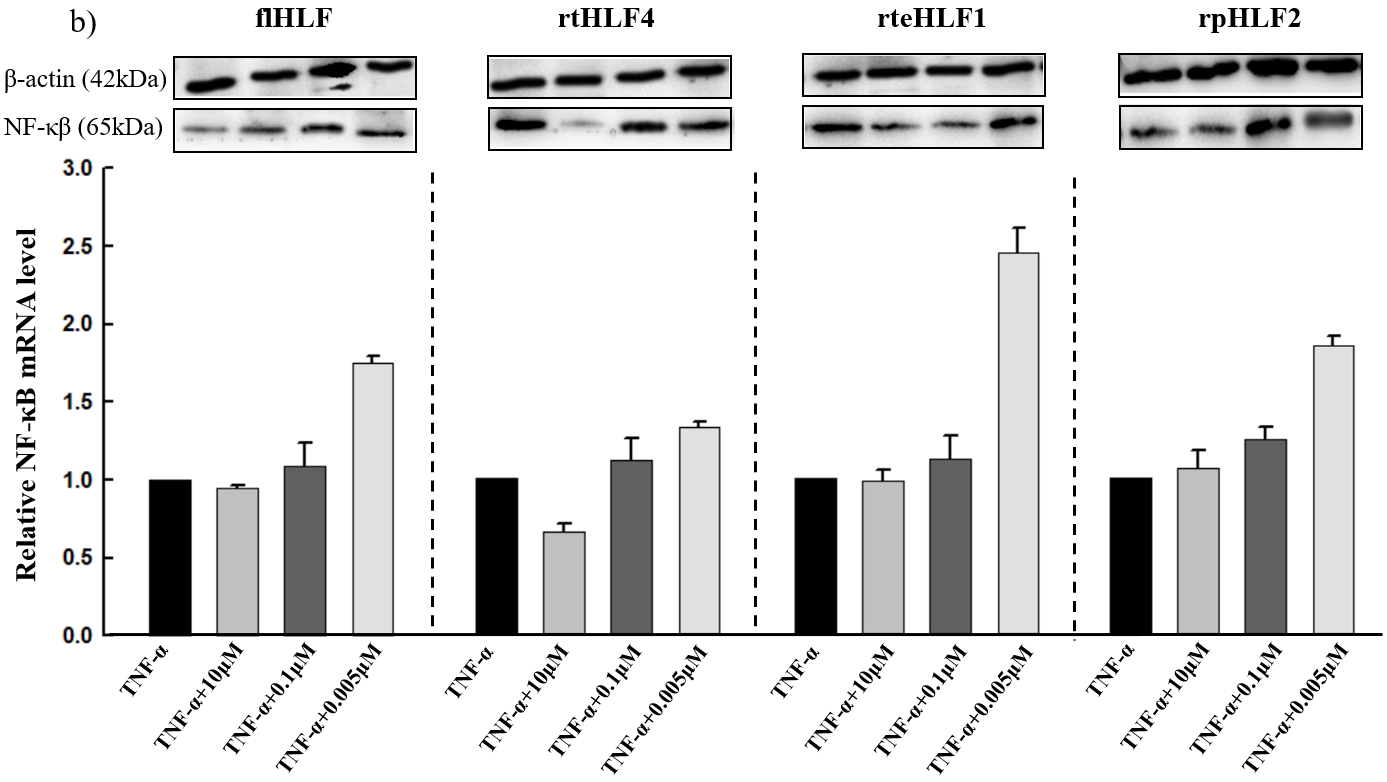


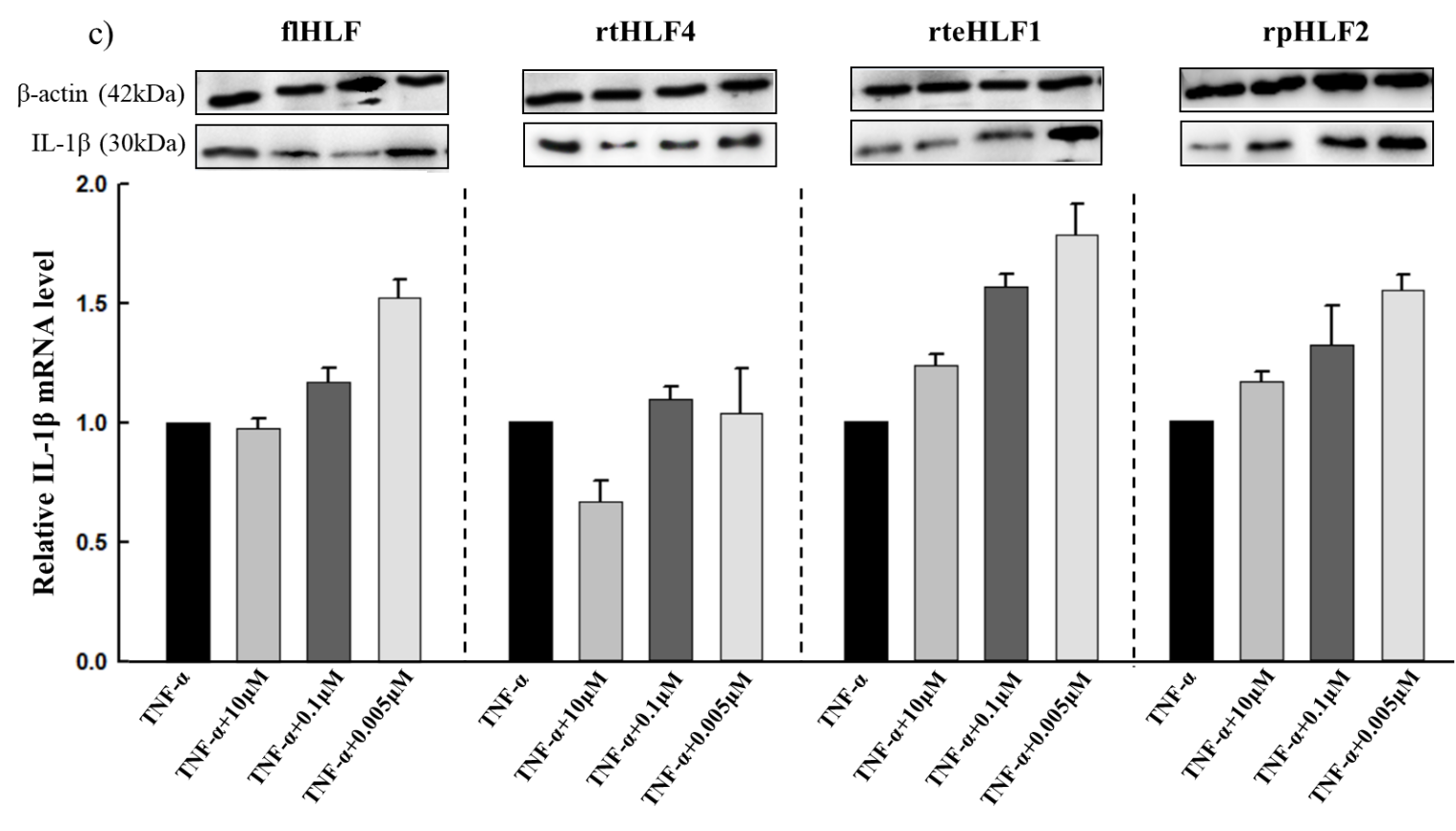


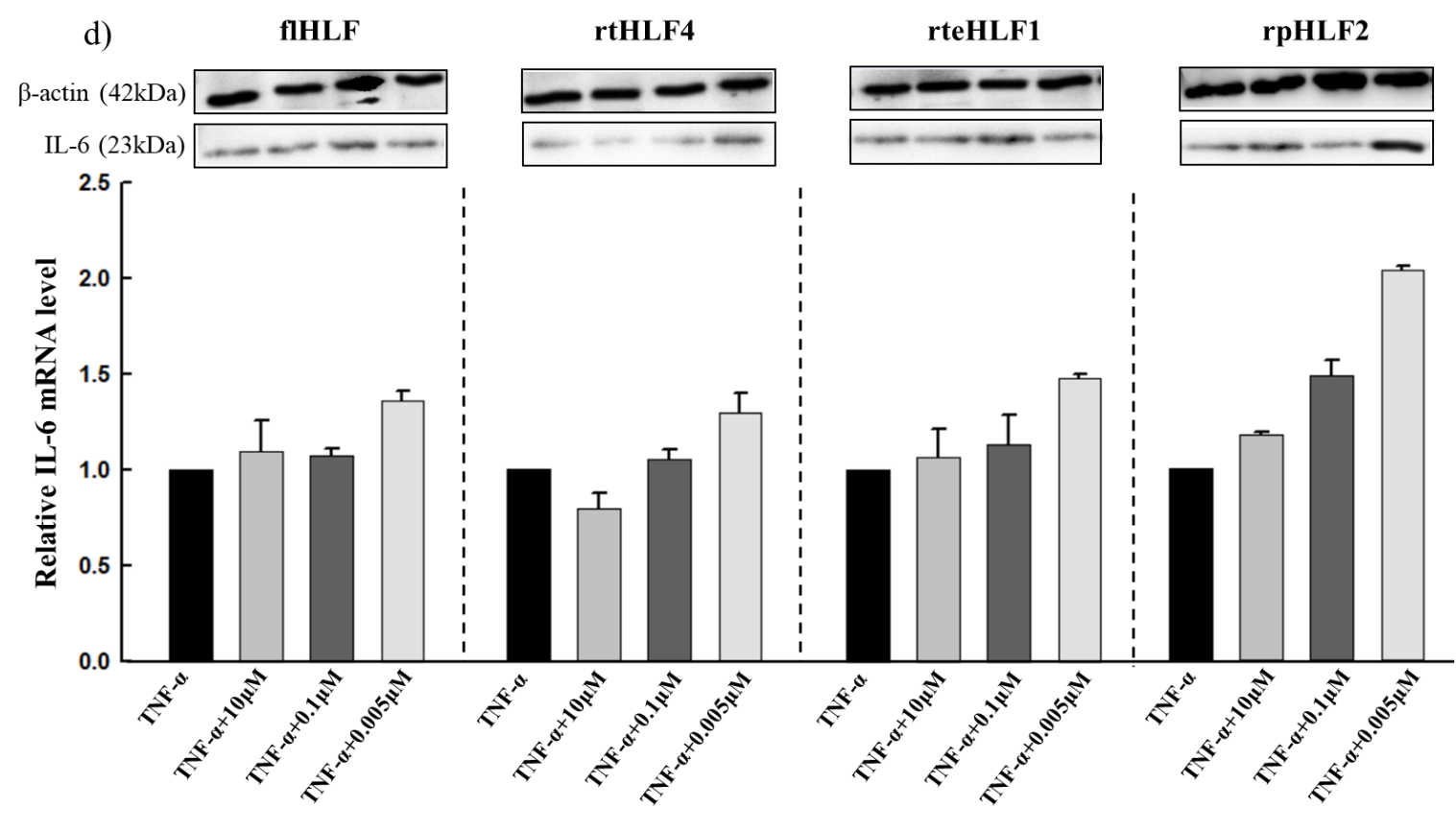


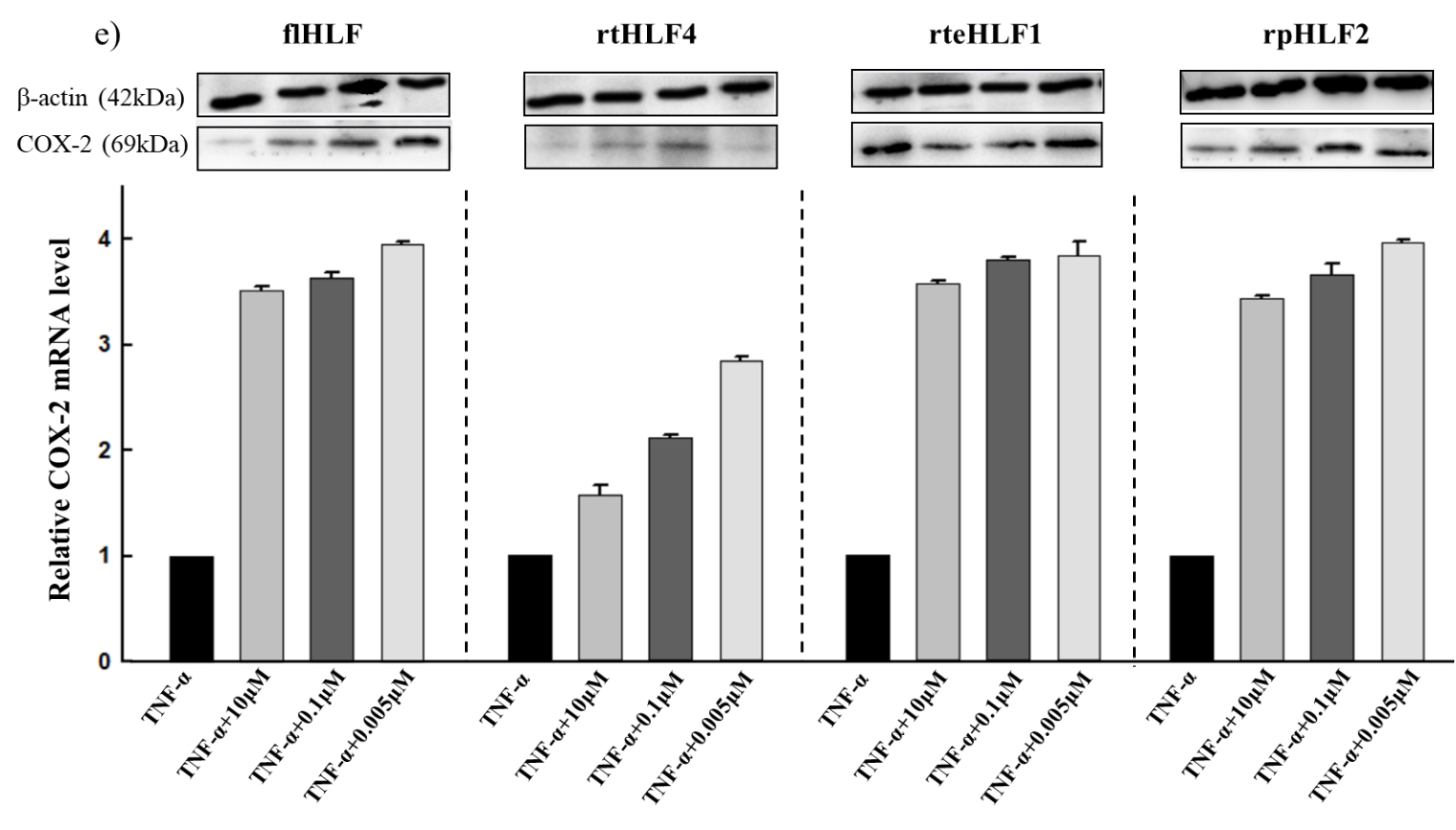


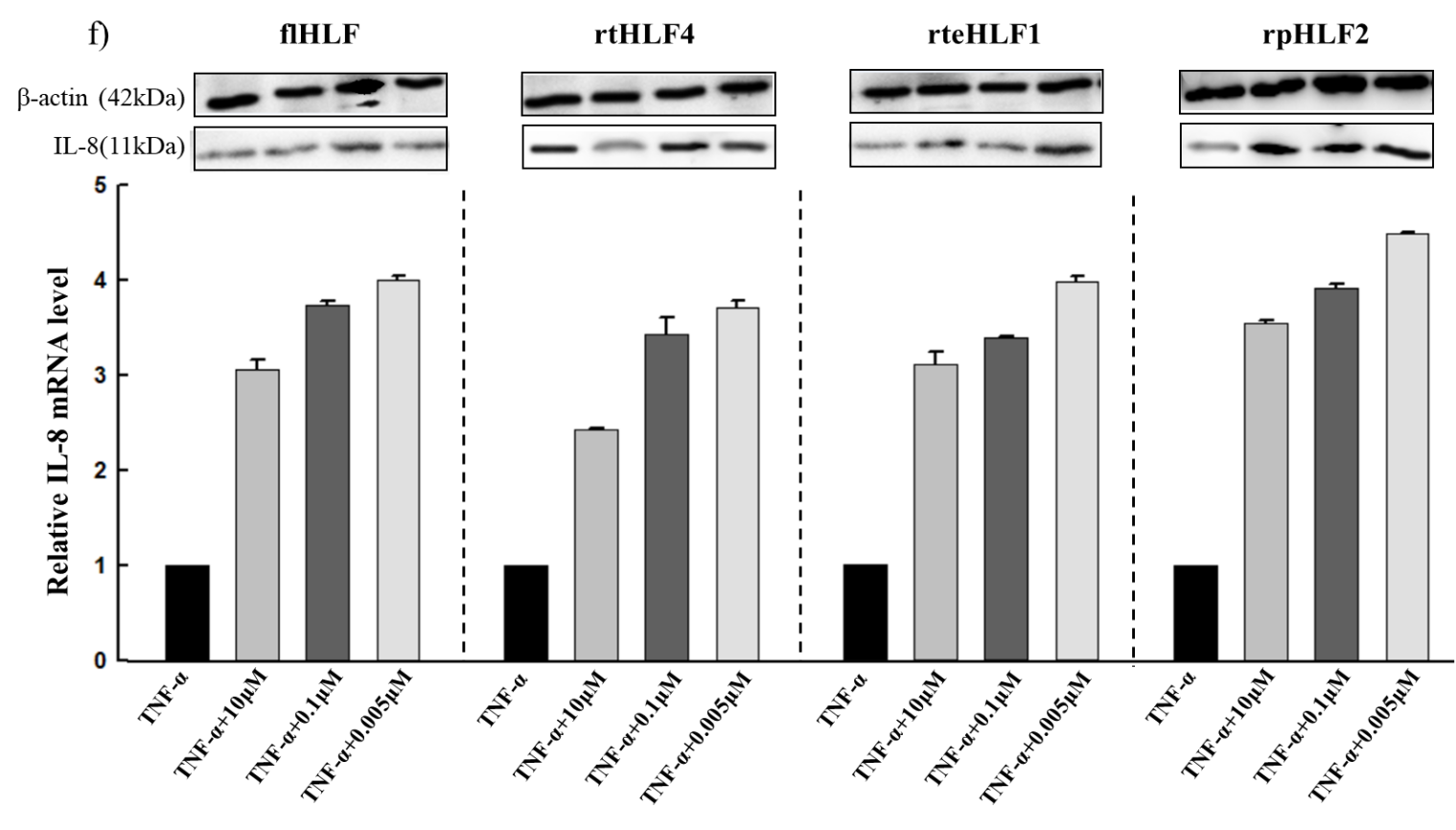


Fig S7: Full length lactoferrin, rtHLF4, rteHLF1 and rpHLF2 with different concentration induce (a) *TNF-α*, (b) *NF-κΒ*, (c) *IL-1β*, (d) *IL-6*, (e) *COX-2*, (f) *IL-8* gene expression and Western blot in human colon adenocarcinoma HT29 cells.
